# Evaluating the effectiveness and cost–benefit of a worksite mental health program among manufacturing workers: A study protocol for a quasi-experimental study

**DOI:** 10.1371/journal.pone.0360481

**Published:** 2026-10-12

**Authors:** Wei Sen Yim, Bala Murali Sundram, Amirah Azzeri, Yin Cheng Lim, Rama Krishna Supramaniam, Suriya Kumareswaran Vallasamy, Siti Nurani Hassan

**Affiliations:** 1 Department of Social and Preventive Medicine, Faculty of Medicine, Universiti Malaya, Kuala Lumpur, Malaysia; 2 Public Health Unit, Department of Primary Care, Faculty of Medicine and Health Sciences, Universiti Sains Islam, Nilai, Seremban, Malaysia; 3 Infectious Disease Unit, Public Health Division, Johor State Health Department, Nilai, Seremban, Malaysia; 4 Consultation Research and Development (CRD), National Institute of Occupational Safety and Health Malaysia (NIOSH), Nilai, Seremban, Malaysia; Universiti Pertahanan Nasional Malaysia, MALAYSIA

## Abstract

**Background:**

Mental health problems lead to the loss of about 12 billion workdays worldwide each year. This creates a major economic burden. In food manufacturing, workers often face various psychosocial stressors. These include high workload, time constraint, repetitive tasks, and low autonomy. These conditions can affect both mental health and work performance. Workplace mental health interventions have shown promising results in mental health improvement. However, most studies come from high-income countries and service sectors. There is still limited evidence from industrial settings in middle-income countries. To address this gap, a structured Worksite Mental Health Program (WMHP) was developed.

**Method:**

This study will employ a quasi-experimental two arm repeated measures design among workers from food manufacturing factories in Malaysia. The intervention group will receive an eight week WMHP including psychoeducation, mindfulness practices, progressive muscle relaxation, and breathing exercises. The comparison group will continue with routine workplace practices and receive a general mental health information pamphlet. Assessments will be carried out at baseline, one month post intervention, and at three months follow up. Primary outcomes include depression, anxiety, stress, and burnout. Secondary outcomes include job satisfaction, absenteeism, presenteeism, and economic outcomes. Changes over time will be analysed using Generalised Estimating Equations (GEE). A cost–benefit analysis (CBA) will also be conducted to assess economic impact.

**Anticipated results:**

The program is expected to improve mental health and work performance, while simultaneously reducing absenteeism and presenteeism. The economic evaluation is expected to demonstrate positive productivity gains associated with the program.

**Conclusion:**

This study will provide evidence on the effectiveness and economic value of a workplace mental health intervention in an industrial setting, informing workplace health strategies in Malaysia and similar contexts.

**Trial registration:**

Thai Clinical Trials Registry (TCTR): TCTR20241217003

## Introduction

Workplace mental health refers to the emotional, psychological, and social well-being of workers in their work environment. This includes their ability to handle stress, perform well and positively impact their organisations. Decent work is defined as safe, secure, and productive employment that provides fair working conditions, supportive management, opportunities for growth, and respect for equality and workers’ rights [1]. Employment and working conditions are widely recognized as key social determinants of health [2]. Recent evidence further demonstrates that good quality work which characterized by fair conditions, job security, and supportive environments promotes better physical and mental wellbeing [3].

Mental health issues at work have become an important public health concern in the last few years. About 60% of the world population is part of the workforce [4], and about 15% of working-age adults have a mental disorder [3,5]. Poor mental health within workers not only compromises individual wellbeing but also incurs significant economic and social costs through absence from work (absenteeism), decrease productivity while working (presenteeism), and premature exit from workforce [6]. Depression and anxiety cause about 12 billion lost workdays around the world every year, which costs the global economy almost $1 trillion each year [7].

Excessive workload, prolonged working hours, limited job control, and poor organisational support are known psychosocial risks at work that can cause occupational mental health problems [8,9]. Evidence indicates that those who work long hours (≥55 hours per week) was associated with an estimated 745,000 deaths from stroke and ischaemic heart disease worldwide in 2016 [10]. These findings show that psychosocial working conditions are closely related to both mental and physical health.

Persistent exposure to psychosocial stressors has been associated with increased symptoms of depression, anxiety and psychological distress, as well as occupational burnout and impaired work functioning [8,9,11,12]. Prolonged exposure to adverse working conditions may also contribute to emotional exhaustion and sleep disturbances [11,13]. In this study, these outcomes will be evaluated as self-reported symptom severity and occupational burnout rather than as clinical psychiatric diagnoses. The DASS-21 will assess symptoms of depression, anxiety and stress, while the OLBI will assess exhaustion and disengagement associated with burnout.

There has been an increasing trend of mental health issues among working-age adults in Malaysia in recent years. This population were still experiencing more depressive and anxiety symptoms according to the National Health and Morbidity Survey (NHMS) 2023 [14]. These trends underscore the need for workplace mental health interventions, as working environment have significant impact on workers’ mental well-being, productivity and economic sustainability. The food manufacturing industry is a very important yet under-studied sector for mental health interventions at work. Food processing contributes a substantial share of Malaysia’s manufacturing output, accounting for about 10% of the sector [15]. In addition, around one-fifth of household consumption expenditure is spent on fast-moving consumer goods (FMCGs), including food and beverages which reflecting the sector’s economic significance [16]. Food manufacturing sector is commonly characterised by shift work, high production demands, repetitive tasks, and deadlines pressure. These conditions are recognised psychosocial risk factors for work-related mental illness [9,17–19]. Emerging international evidence suggests that workers in this sector experience both physical and psychological burdens. A study conducted among Korean food manufacturing workers indicated a significant correlation between adverse work environments, prolonged working hours, and the prevalence of sleep-related problems, musculoskeletal pain, and burnout [20]. According to a Malaysian systematic review, prolonged working hours, poor working conditions, insufficient organisational support, and low job control were listed as key contributors to depression, anxiety, and stress among food industry workers [21].

At policy level, international frameworks are starting to see mental health at work as an important component of occupational safety and health. World Health Organization (WHO) and International Labour Organization (ILO) have emphasised the need of psychosocial risks prevention, mental health promotion at work, and support for workers with mental health problems through actions at the organisational, managerial, and individual levels [3,6]. These recommendations are consistent with evidence from studies demonstrating the effectiveness of comprehensive workplace mental health interventions [9,22,23]. The Occupational Safety and Health Master Plan 2025 (OSHMP25) and initiatives from the Department of Occupational Safety and Health and the Social Security Organisation in Malaysia are testaments to these principles [24].

Beyond demonstrating effectiveness, economic evidence has become more important in informing employer and policy decisions related to workplace mental health interventions [25,26]. Findings from systematic reviews of economic evaluations indicate that such programs can produce favourable economic outcomes, largely through productivity improvements associated with reductions in absenteeism and presenteeism. Nevertheless, the strength and long-term sustainability of these economic benefits vary across studies [27,28]. Much of the available evidence originates from research conducted in high income countries and is largely based on service sector or white-collar occupational settings. In contrast, there remains limited economic evidence from middle income countries and from industrial work environments, where working conditions and organisational structures may differ substantially [29,30].

This evidence gap is particularly apparent in industrial sectors, including manufacturing, where workplace mental health interventions remain under-studied and where integrated evaluations combining effectiveness and economic outcomes are uncommon. Within manufacturing, evidence specific to the food manufacturing sector is especially limited, despite its high job demands and exposure to psychosocial risks. The absence of integrated cost–benefit analysis (CBA) in such settings may constrain the scalability and sustainability of workplace mental health interventions in cost-sensitive industries.

This study aims to evaluate both the effectiveness and the cost–benefit of the Worksite Mental Health Program (WMHP) implemented in food manufacturing factories in Malaysia. The study integrates assessment of mental health outcomes with an economic evaluation to provide a more comprehensive understanding of the program’s impact. By doing so, the protocol seeks to generate evidence that is relevant for decision making and may inform the adoption, scale-up, and long-term sustainability of workplace mental health interventions in industrial settings.

## Materials and methods

### Study design

This is a two-arm quasi-experimental study designed to evaluate the effectiveness of a WMHP among workers in a food manufacturing setting in Malaysia. The study compares an intervention factory receiving the WMHP in addition to routine workplace practices with a comparison factory continuing routine workplace practices and receiving a general mental health information pamphlet. Quasi-experimental design is commonly used in workplace intervention research where randomisation is infeasible due to operational, production, or organizational constraints [31–33]. Such approach enables evaluation of real-world effectiveness while maintaining methodological rigour with a comparison group and repeated outcome measurements.

The study will include one intervention factory and one comparison factory. Intervention status will therefore be aligned with factory, and workers will be nested within their respective factory. Differences between the two groups may consequently reflect both the WMHP and factory-specific characteristics.

Data will be collected at three timepoints:

1. Baseline (T0) – prior to intervention
2. Post-intervention, 1 month (T1)
3. Post-intervention, 3 months (T2)

The schedule of enrolment, intervention, and assessments is presented in Fig 1 [34]. Repeated assessments across multiple follow-up timepoints are recommended in psychosocial and workplace mental health interventions to capture both immediate and short-term sustained effects on psychological wellbeing and productivity [3,35]. This design allows assessment of both immediate and sustained effects of the WMHP on psychological wellbeing and workplace productivity.

**Fig 1:**
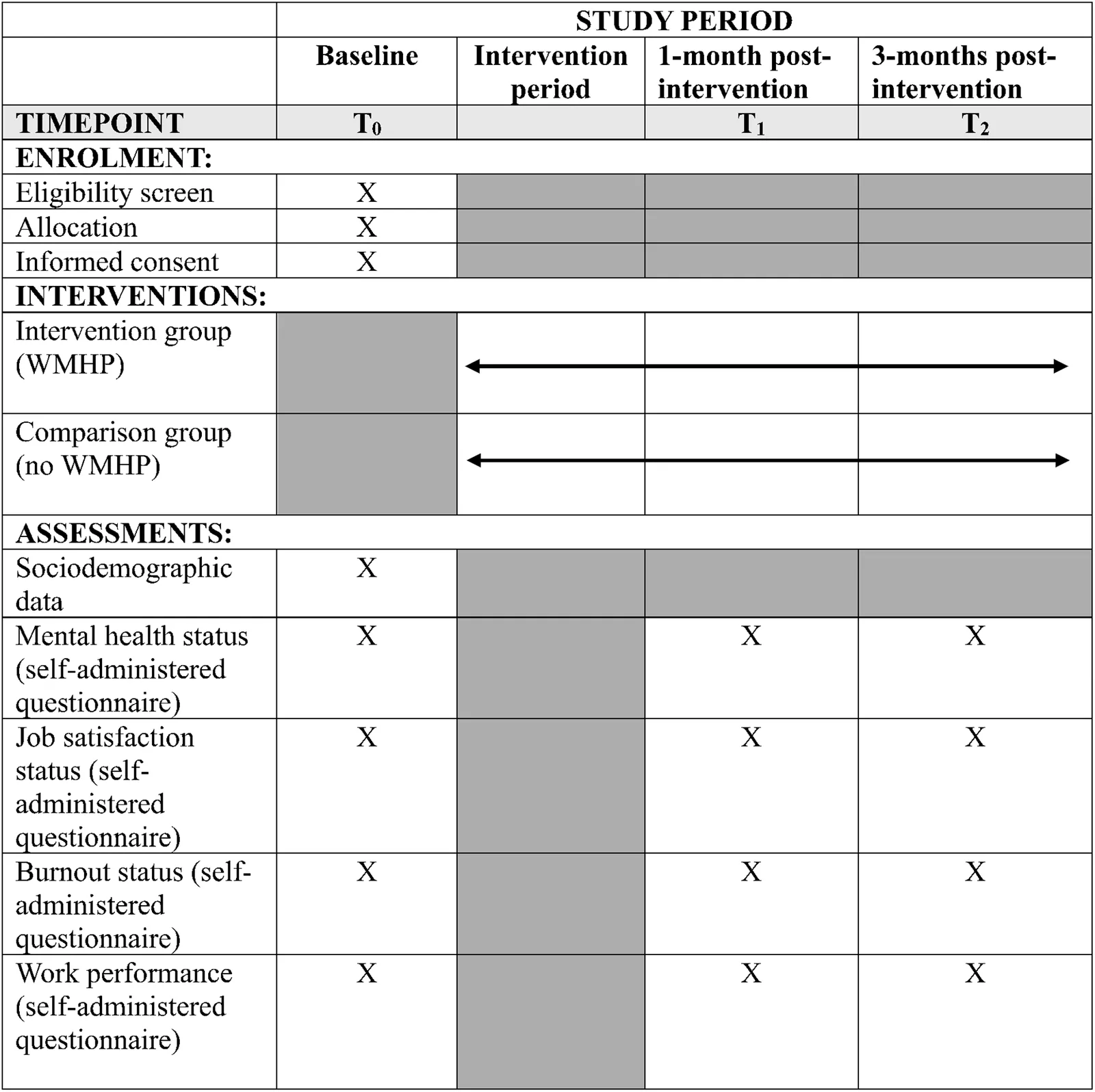
SPIRIT schedule of enrolment, intervention and assessments.

### Conceptual model

This study is based on well-established occupational models of job stress and health, which view mental health outcomes as the consequence of extended exposure to psychosocial stressors in the workplace. The impact of these stressors is perceived to operate through individual stress responses, coping capacity, and behavioural adaptation within this perspective [36–38]. Within this model, WMHP targets both individual and contextual determinants of worker mental health. It aims to strengthen personal coping resources and fostering supportive organisational practices. According to this model, work environment is not only a determinant of physical safety but also a critical psychosocial ecosystem that shapes workers’ psychological wellbeing, motivation, and productivity.

The WMHP is designed to interrupt this stress–health–productivity pathway by providing structured interventions within the work environment. There are 3 components within the program, includes psychoeducation to improve mental health literacy, mindfulness-based practices to enhance emotional awareness and attention regulation, and relaxation activities such as progressive muscle relaxation and breathing exercises to reduce physiological stress activation [37–39]. The purpose of these components is to improve self-regulation, resilience, and adaptive coping. In doing so, it enables workers to manage stressors more effectively and recover more rapidly. Ultimately, the intervention addresses both the proximal determinants of psychological strain and the distal outcomes that affect organisational performance.

In manufacturing settings, workers are frequently exposed to high job demands, repetitive and monotonous tasks, strict production targets, and shift schedules that reduce recovery opportunities and limit decision latitude [9,18,38]. This activates a series of psychological and physiological stress responses, such as heightened arousal, emotional exhaustion, and impaired cognitive functioning. When these responses persist without adequate rest and coping mechanisms, they may contribute to symptoms of anxiety and depression, together with occupational burnout and impaired work functioning. From an organisational perspective, these effects always manifest as reduced engagement, higher absenteeism, presenteeism, and productivity loss. Eventually leading to significant indirect economic costs [40,41].

Consistent with the results of meta-analytic evidence, it is anticipated that improvements in psychological well-being and reductions in burnout will result in increased levels of work engagement, greater job satisfaction, and enhanced overall work performance. Simultaneously, these changes may result in decreased absenteeism and presenteeism, as well as reduced dependence on healthcare services [35,38,40,41]. These dual psychological and economic benefits provide a clear rationale for evaluating the effectiveness and cost benefit of the WMHP in food manufacturing settings where psychosocial risks are often overlooked compared to physical hazards.

For evaluation purposes, this conceptual framework is operationalised using an Input–Process–Output (IPO) logic model [42,43]. Fig 2 illustrates the conceptual framework of the WMHP based on the IPO model, outlining the logical linkage between program resources, intervention processes, and resultant psychological, work-related, and economic outcomes.

**Fig 2:**
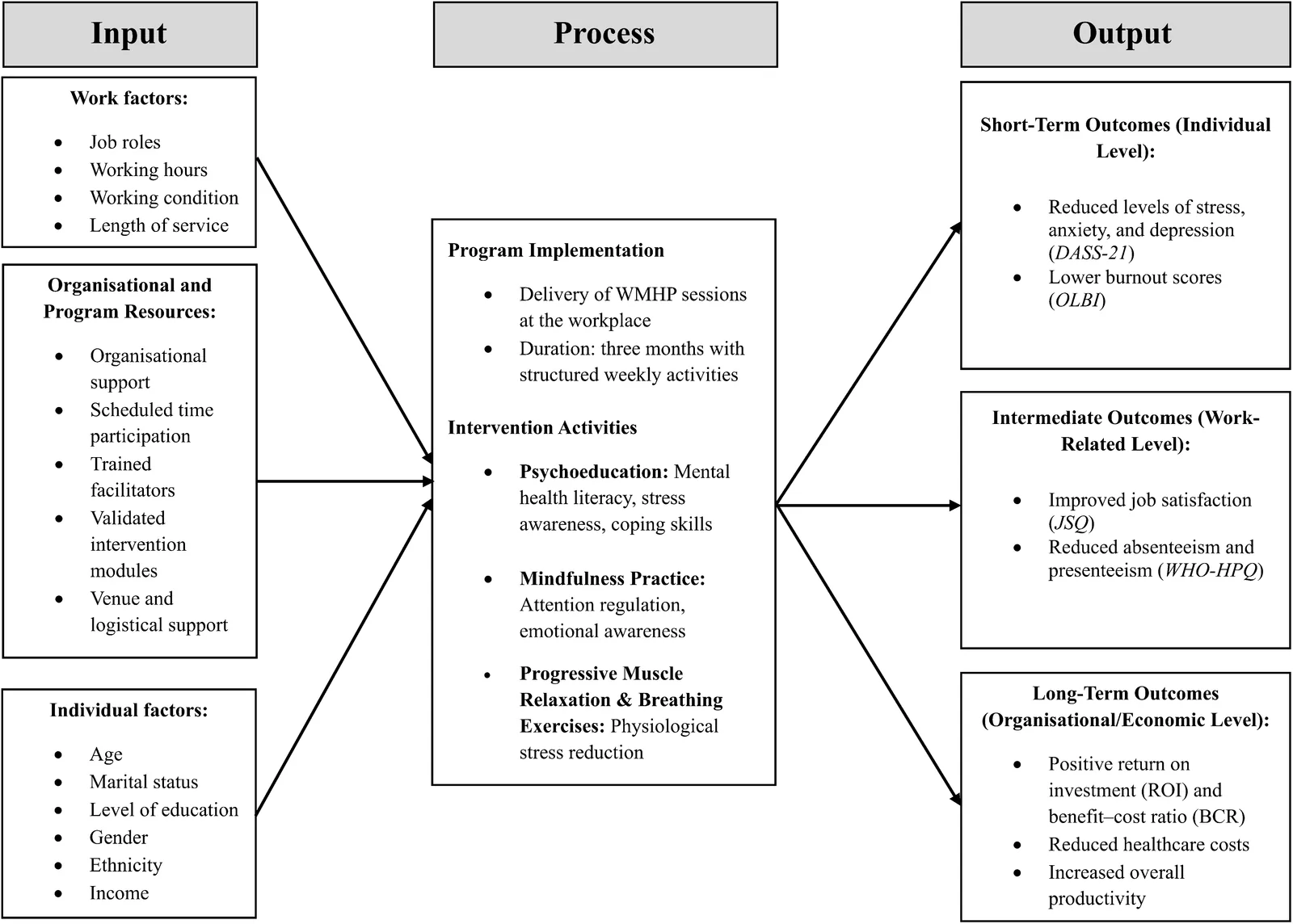
Conceptual framework of the WMHP based on the Input–Process–Output (IPO) Model.

The IPO model provides a structured framework in understanding how programme components and its mechanisms are expected to produce measurable outcomes. As for the inputs, it consists of organisational and program resources such as trained facilitators, intervention materials, management support, and allocated time, together with relevant individual and workplace factors that may influence program implementation. These inputs support the intervention processes that include series of activities such as psychoeducation sessions, mindfulness practice, progressive muscle relaxation, and breathing exercises. These activities are designed to promote stress management, coping, and emotional regulation among participants. The final outputs occur across several levels. The program is expected to reduce symptoms of stress, anxiety, depression, and burnout while improving psychological well-being in short term. Intermediate outcomes include increased job satisfaction and reductions in absenteeism and presenteeism. In the long term, these enhancements may result in increased productivity and reduced healthcare utilisation, thereby generating economic benefits for the organization. By linking individual level mechanisms with organisational outcomes, this layered logic model supports an integrated evaluation of programme effectiveness and economic return within a single analytical framework [38,44].

### Study setting

The study will be conducted in two food manufacturing factories located in Negeri Sembilan, Malaysia. Food manufacturing factories that indicate their willingness to participate will first be compiled into a list. Two factories will then be selected from this list using simple random sampling so that each participating factory has an equal chance of being chosen for the study. The WMHP will be delivered by trained members of the research team using the standardised intervention protocol and materials.

### Study participants

The study participants will comprise of full-time Malaysian workers working in two food manufacturing factories located in Negeri Sembilan. Eligible participants should age between 18 and 60 years old who have been employed at the factory for at least one year, ensuring adequate familiarity with the work environment. No minimum formal educational qualification will be required. However, participants must be able to read and understand either Bahasa Melayu or English sufficiently to review the participant information sheet, provide written informed consent, and complete the self-administered questionnaires. A standardised briefing will be provided before enrolment, during which potential participants may request clarification. Research personnel may provide procedural clarification during questionnaire administration but will not interpret individual questionnaire items or influence participants’ responses.

Workers with a diagnosis of psychiatric disorder under active treatment will be excluded. The same goes for workers with chronic illnesses that may independently influence mental health or work performance, such as heart disease, chronic kidney disease, or cancer. Pregnant workers will not be included due to potential pregnancy related physiological and psychological changes that may affect stress or work performance. In addition, workers who have participated in any stress management or mental health program within the past year will be excluded to prevent potential confounding effects on the outcomes of the WMHP.

### Participant recruitment and sampling

Eligible workers in each selected factory will be identified based on the inclusion and exclusion criteria. Simple random sampling will be used to recruit participants from the eligible worker list provided by the human resource department to minimise selection bias and ensure equal probability of participation. Random selection will be conducted using computer-generated random numbers.

All selected participants will be invited to go through workplace briefings and written information sheets. Participation will be voluntary, and informed consent will be taken prior to data collection.

Fig 3 presents the planned participant flow for the WMHP study, showing the number of workers assessed for including eligibility assessment, randomly selection within factory, enrolment into the intervention or comparison group according to factory, follow-up and analysis. The diagram was adapted according to applicable SPIRIT reporting principles for a non-randomised quasi-experimental study.

**Fig 3:**
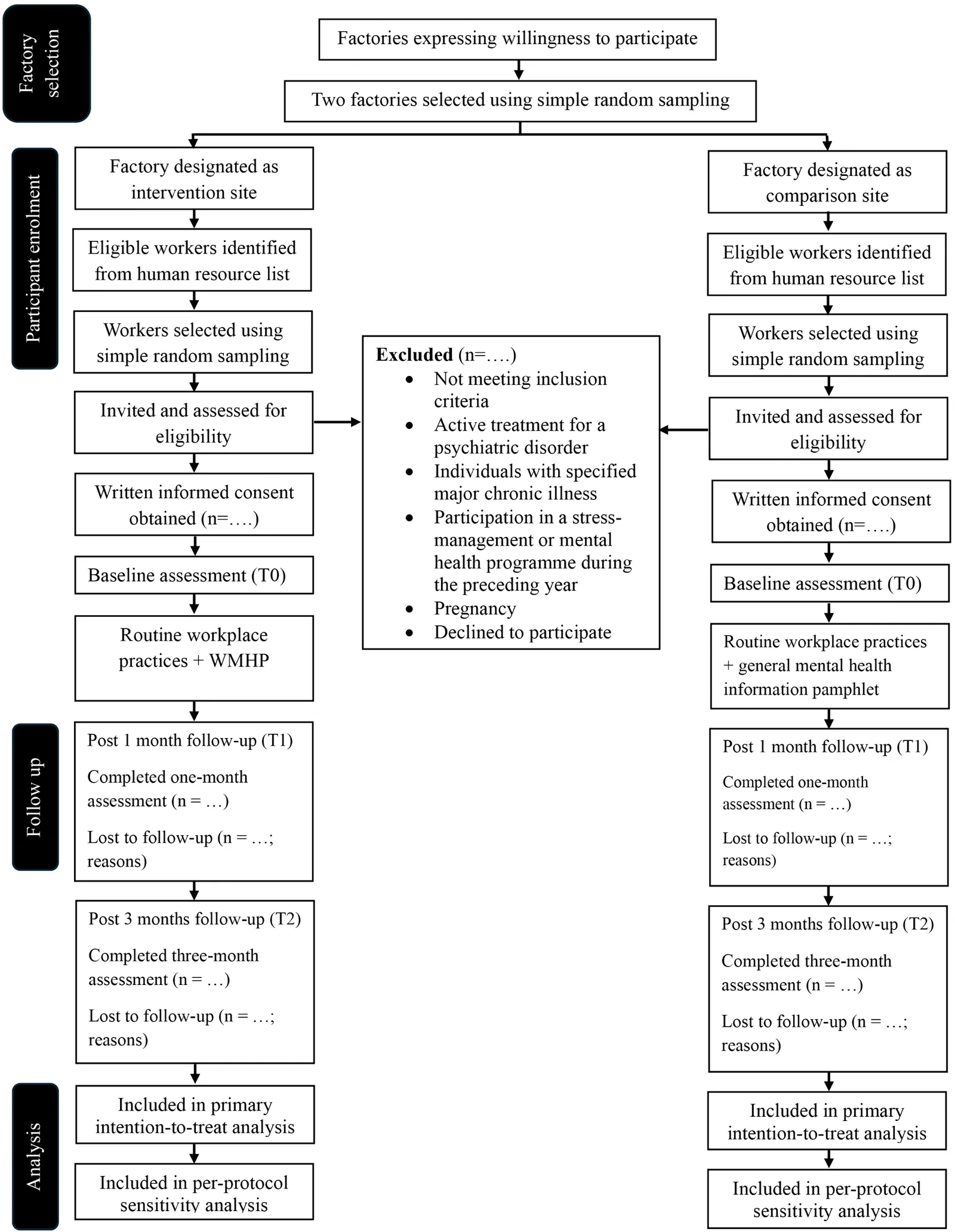
Participant flow diagram of the WMHP study.

### Sample size

The sample size for this quasi-experimental study was estimated with reference to previous workplace mental health and occupational intervention research that adopted similar repeated-measures designs analysed using Generalised Estimating Equations (GEE). Previous studies demonstrated moderate improvements in psychological wellbeing and work performance following psychoeducational and mindfulness-based programs [45,46], while meta-analytic evidence shown small to moderate effects of workplace interventions on productivity and mental health outcomes [47]. The sample size was calculated using G*Power version 3.1 for an ANOVA repeated-measures within–between interaction. Assuming a moderate effect size of Cohen’s d = 0.40 (equivalent to f = 0.20), a significance level of 0.05 and 80% statistical power, the minimum required sample size was 82 participants. Allowing for approximately 20% attrition, the recruitment target was increased to 100 participants, with approximately 50 participants per group and a 1:1 recruitment ratio.

The sample size calculation was conducted at the individual-participant level and did not incorporate an intraclass correlation coefficient (ICC) or design-effect adjustment for clustering within factories. As the study includes only two factories, with one factory in each study arm, reliable estimation of the ICC and conventional cluster-adjusted power is limited. The nominal power may therefore overestimate the effective power to distinguish intervention-related changes from factory-specific influences.

### Overview and study duration

This study comprises three sequential phases conducted over an estimated period of 24–30 months. Phase 1 involves the systematic development and validation of the WMHP, including needs assessment, intervention mapping, expert content validation, and pilot testing. Phase 2 focuses on the implementation of the WMHP and the evaluation of its effectiveness using a quasi-experimental design with repeated outcome measurements. Phase 3 involves a CBA conducted alongside and following the effectiveness evaluation to assess the economic value of the WMHP from an employer perspective.

The present protocol describes the methodological approach for the implementation, effectiveness evaluation, and economic analysis of the WMHP within food manufacturing settings in Malaysia.

Fig 4 provides an overview of the three sequential phases of the WMHP, showing the progression from program development and validation (Phase 1) to implementation and effectiveness evaluation (Phase 2), followed by the CBA (Phase 3).

**Fig 4:**
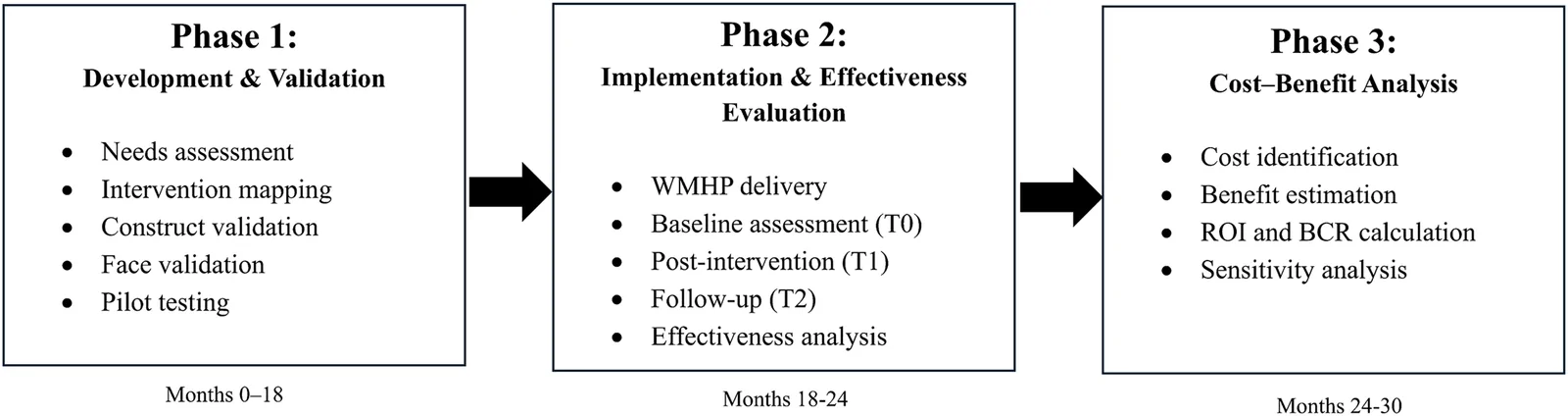
Study phases of the WMHP.

At the time of initial manuscript submission, participant recruitment and data collection had not commenced. Recruitment was completed in June 2026, data collection is expected to be completed by October 2026, and final study results are anticipated by June 2027.

### Phase 1: development and validation of the WMHP

This phase focused on the development and validation of the WMHP using the Implementation Mapping framework. It started with a needs assessment to identify key psychosocial risk factors, behavioural determinants, and organisational influences that affecting mental health among food manufacturing workers. Based on these findings, the program objectives and change matrices were developed.

The intervention was designed using evidence-based strategies and delivered as a multi component program. It included psychoeducation, mindfulness practice, progressive muscle relaxation, and breathing exercises. To facilitate the program’s implementation, the intervention materials were created in culturally appropriate formats. They consisted of video resources, guided practice scripts, a digital flipbook, and presentation slides.

The content validity was assessed by experts in occupational health, public health, and mental health. Then followed by face validation among workers to ensure clarity and feasibility. Workers were involved during programme development through the needs-assessment and face-validation processes, and their feedback informed the clarity, feasibility and contextual appropriateness of the intervention materials and delivery approach. Revisions were made based on expert and participant feedback. Beyond this developmental involvement, workers will not have a formal role in study governance, data analysis, interpretation or preparation of study reports.

### Phase 2: implementation and effectiveness evaluation of the WMHP

Phase 2 focuses on the implementation of the WMHP and the evaluation of its effectiveness using a quasi-experimental, two-arm, pre–post study design conducted in both factories. The WMHP will be implemented in one factory designated as the intervention group, while the comparison factory will continue routine workplace practices and receive a general mental health information pamphlet.

The WMHP will be delivered according to a standardised implementation protocol to ensure consistency across participants. Validated self-administered questionnaires in Bahasa Melayu and English will be used to evaluate effectiveness outcomes at three time points: baseline (T0), one month post intervention (T1), and three months post intervention (T2). Participants will complete the questionnaires in their preferred language. This approach enables evaluation of both immediate and short-term effects of the WMHP on psychological wellbeing and work-related outcomes. The data obtained during this phase will serve as the primary basis for both the effectiveness analysis and the economic evaluation conducted in Phase 3.

### Phase 3: CBA of the WMHP

The CBA will identify, measure and value the costs and benefits of the WMHP from the employer perspective over the three-month study follow-up period. Costs will include direct programme and implementation resources, such as facilitator time, intervention materials and administrative resources, together with the indirect opportunity cost of paid worker time spent attending the intervention and reinforcement sessions. Costs incurred solely for research purposes, including research data collection and statistical analysis, will be excluded.

Monetised benefits will include productivity gains associated with changes in absenteeism and presenteeism, reductions in objectively recorded medical-certificate or sick-leave days, and healthcare savings associated with changes in panel clinic utilisation. Productivity-related benefits will be valued using prespecified wage-based approaches, while healthcare-related benefits will be valued using relevant clinic unit costs. Outcomes not directly measured, including staff turnover and aggregate factory production output, will not be monetised.

The primary economic analysis will estimate incremental benefits based on the difference in changes between the intervention and comparison factories over the study period, rather than relying solely on within-factory changes. Economic outcomes will be reported as net benefit, benefit–cost ratio (BCR) and return on investment (ROI). Discounting will not be applied because the time horizon is less than one year. Sensitivity analyses will examine alternative assumptions regarding programme costs, wage rates, healthcare unit costs, productivity gains and the proportion of observed benefits attributable to the intervention.

### Intervention: Worksite Mental Health Program (WMHP)

A structured, multi-component intervention is developed to address common psychosocial risk factors and mental health promotion among food manufacturing workers. The program integrates individual-level mental health promotion, stress management strategies, and workplace-relevant psychosocial approaches, tailored to the operational context of food manufacturing environments.

The WMHP will be delivered through an initial instructor-led session, followed by scheduled reinforcement sessions conducted at two-weekly intervals to support skill consolidation and continued engagement. Guided self-practice materials will also be provided to participants to facilitate ongoing application of the techniques within routine workplace settings. Attendance at the initial WMHP session and each reinforcement session will be recorded using attendance logs to monitor programme adherence.

The intervention consists of the following component:

- Psychoeducation that aimed at improving mental health literacy and increasing awareness of workplace stress while introducing practical coping strategies
- Stress management techniques, including mindfulness-based practices, progressive muscle relaxation, and breathing exercises
- Development of skills that strengthen emotional regulation and support workers in responding more effectively to work-related stress

The WMHP will be delivered in addition to the routine workplace practices already available at the intervention factory.

### Comparison group

Participants in the comparison factory will continue with routine workplace practices and will receive a general mental health information pamphlet. The pamphlet provides basic mental health information but does not include the facilitated psychoeducation, guided mindfulness practice, progressive muscle relaxation, breathing exercises or scheduled reinforcement activities provided through the WMHP. This minimal-information comparison condition provides participants with access to basic mental health information while maintaining a clear distinction from the structured WMHP. The use of separate factories for the intervention and comparison groups is expected to minimise direct cross-arm contamination. However, exposure of comparison participants to similar mental health information through supervisors, coworkers or other non-study sources cannot be completely excluded.

### Outcome measures

#### Primary outcomes.

The primary outcomes are symptoms of depression, anxiety and stress, assessed using the Depression Anxiety Stress Scales–21 (DASS-21), and occupational burnout, assessed using the Oldenburg Burnout Inventory (OLBI). The OLBI evaluates the two dimensions of exhaustion and disengagement.

#### Secondary outcomes.

Secondary outcomes include job satisfaction, absenteeism, presenteeism, and economic evaluation indicators. Job satisfaction will be assessed using the Job Satisfaction Questionnaire (JSQ), while self-reported absenteeism and presenteeism will be evaluated using the World Health Organization Health and Work Performance Questionnaire (WHO-HPQ). In addition, objective absenteeism will be documented using medical certificate (MC) and sick leave records obtained from panel clinic data. Clinic utilisation will also be measured to assess potential changes in healthcare use associated with the intervention.

Economic outcomes will be examined as part of the CBA of the WMHP. Productivity related indicators and healthcare utilisation data obtained during the study will contribute to the estimation of economic benefits associated with the intervention.

### Measurement instruments

The DASS-21, OLBI, JSQ and WHO-HPQ will be administered as self-completed questionnaires in Bahasa Melayu and English. Participants will complete their preferred language version. Published psychometric evidence supporting the original English instruments and the relevant Bahasa Melayu versions is described below.

The DASS-21 will be used to assess symptoms of depression, anxiety and stress. It comprises 21 items distributed equally across three seven-item subscales. Each item is rated on a four-point response scale, and subscale scores will be calculated according to the recommended scoring procedure. Higher scores indicate greater symptom severity. The original English DASS-21 has demonstrated satisfactory construct validity, while the Bahasa Melayu version has undergone translation and psychometric validation in a Malaysian population [48,49].

The OLBI will be used to assess occupational burnout across the dimensions of exhaustion and disengagement. The instrument comprises 16 positively and negatively worded items, with eight items in each dimension, rated on a four-point response scale. Relevant items will be reverse-scored before the exhaustion and disengagement scores are calculated, with higher scores indicating greater burnout. The English version has demonstrated satisfactory construct validity, while a Bahasa Melayu translation has undergone face-validity, factor-structure and internal-consistency assessment in Malaysia [50,51].

The JSQ will be used to assess job satisfaction across 34 items and eight domains: teamwork, leadership, rewards and recognition, empowerment and participation, training and development, working hours, communication, and working conditions. Items are rated on a five-point Likert scale, with higher scores indicating greater job satisfaction. The JSQ was developed and psychometrically validated in Malaysia among healthcare workers, demonstrating satisfactory content validity, factor structure and internal consistency across its eight domains [52].

The short form of the WHO-HPQ will be used to assess self-reported absenteeism and presenteeism. Absenteeism will be derived from the reported number of workdays missed during the specified recall period. Presenteeism will be assessed using the absolute work-performance rating, ranging from 0, representing the worst possible performance, to 10, representing the best possible performance. Higher ratings indicate better perceived work performance. The original WHO-HPQ was developed to evaluate health-related workplace attendance and performance, while its Bahasa Melayu version has undergone cross-cultural adaptation and psychometric assessment among Malaysian workers [53,54].

In addition to the self-reported measures, objective data on clinic utilisation and MC or sick leave records will be obtained from panel clinic administrative databases for participating workers. These administrative data will primarily be used to support the economic evaluation component of the study.

### Data collection procedures

Data will be collected at three assessment timepoints: baseline prior to intervention implementation (T0), one month post intervention (T1), and three months post intervention (T2). These timepoints are selected to capture the immediate and short-term changes in psychological wellbeing, work-related outcomes, and economic indicators associated with the intervention.

Both intervention and comparison group participants will be asked to complete validated self-administered questionnaires at each assessment timepoint. Questionnaires will be administered during scheduled workplace data collection sessions and completed using unique study identifiers to ensure confidentiality. Members of the research team will be present to provide procedural clarification where required, without influencing participants’ responses.

Work performance outcomes related to absenteeism and presenteeism will be assessed using the World Health Organization Health and Work Performance Questionnaire. These measures will contribute to both the evaluation of intervention effectiveness and the subsequent economic analysis.

Clinic utilisation data will be obtained from panel clinic administrative records for participating workers, including the number of clinic visits and associated MC or sick leave issuance during the study period. These data will be retrieved in de identified form in collaboration with panel clinics and factory management and will be used to quantify healthcare utilisation and objective absenteeism for the CBA.

Data collected during Phase 2 will constitute the primary dataset for the evaluation of intervention effectiveness and will also serve as key inputs for the CBA conducted in Phase 3, enabling integration of mental health outcomes with productivity and healthcare utilisation indicators.

Participants who discontinue or deviate from the intervention but do not withdraw consent for follow-up data collection will be encouraged to complete the scheduled follow-up assessments. Follow-up assessments will be coordinated with the participating factories, and reminders will be provided to support participant retention. Reasons for loss to follow-up, where available, will also be documented.

### Cost–Benefit Analysis (CBA)

A CBA will be conducted as Phase 3 of the study to evaluate the economic value of the WMHP from an employer perspective. The analysis will be performed alongside and following the effectiveness evaluation, using outcome data collected during Phase 2.

### Perspective and time horizon

The CBA will be conducted from the employer perspective over the three-month study follow-up period, focusing on costs and benefits that are directly relevant to the participating food manufacturing factories. The primary time horizon will correspond to the study follow-up period, covering baseline, one-month post-intervention, and three-month post-intervention assessments. Outcome and economic data for the intervention and comparison factories will be collected over corresponding calendar periods to minimise differential seasonal influences. Longer-term costs and benefits beyond the observation period will not be modelled. Discounting will not be applied because all costs and benefits will occur within one year.

### Cost identification and measurement

Programme costs will include development resources relevant to future implementation, facilitator time, intervention materials, administrative and coordination resources, and the opportunity cost of paid worker time spent attending the initial intervention and reinforcement sessions. Venue and equipment costs will be included where they are incurred specifically for programme delivery. Costs arising solely from research activities, including research questionnaire administration, research data collection, statistical analysis, manuscript preparation and dissemination, will be excluded.Cost data will be obtained from project records, administrative documents, and applicable wage rates. Resource quantities and corresponding unit costs will be documented separately, and all costs will be expressed in Malaysian Ringgit for the relevant study year.

### Benefit identification and valuation

Benefits will be estimated from changes in self-reported absenteeism and presenteeism, objectively recorded MC or sick-leave days, and panel clinic utilisation observed during Phase 2. The primary benefit estimates will be based on the incremental change in the intervention factory relative to the comparison factory over the same study period, rather than solely on before-and-after changes within the intervention factory. Where applicable, group-by-time estimates from the effectiveness analysis will be used to inform the magnitude of intervention-associated changes.Absenteeism-related productivity benefits, including reductions in MC or sick-leave days, will be valued using the applicable average daily wage. Presenteeism-related productivity gains will be monetised using a prespecified wage-based valuation approach applied to the WHO-HPQ results. Healthcare-related savings will be estimated by multiplying changes in panel clinic utilisation by the applicable unit cost per clinic visit. Outcomes that are not directly measured, including employee turnover, replacement costs and aggregate factory production output, will not be monetised.

### Economic outcome measures

Economic outcomes will be summarised using standard cost–benefit metrics, including net benefit, BCR, and ROI. Net benefit will be calculated as the difference between total monetised benefits and total program costs. The BCR will be computed as the ratio of total benefits to total costs, while ROI will be expressed as the percentage return per unit of investment. A BCR greater than 1 indicates that the economic benefits exceed the program costs, suggesting that the intervention yields a positive economic return.

The calculations will be based on the following formulas:


$$\begin{array}{c} Net\;Benefit\;=Total\;Benefits\;-Total\;Costs \end{array}$$

$$\begin{array}{c} Benefit-Cost\;Ratio\;(BCR)=Total\;Benefits\;÷Total\;Costs \end{array}$$

$$\begin{array}{c} Return\;on\;Investment\;(ROI)\;(\%)=(Total\;Benefits\;-Total\;Costs)÷Total\;Costs\times \text{1}\text{0}\text{0} \end{array}$$

### Sensitivity analysis

Sensitivity analyses will assess the robustness of the economic findings under alternative assumptions regarding programme costs, wage rates, productivity gains and panel clinic unit costs. To examine uncertainty arising from factory-specific, seasonal or wider economic conditions, conservative scenarios will reduce the estimated incremental productivity benefits attributed to the WMHP by 25% and 50%. Additional scenarios will exclude presenteeism-related benefits and restrict monetised benefits to objectively recorded MC or sick-leave days and panel clinic utilisation. The resulting changes in net benefit, BCR and ROI will be reported.

### Statistical analysis

Data analyses of this study will be conducted using IBM SPSS Statistics (version 29.0) or equivalent statistical software. The primary effectiveness analyses will follow an intention-to-treat principle, whereby participants will be analysed according to their original intervention or comparison factory, irrespective of their level of adherence to the WMHP. All available outcome observations will be included in the primary analyses. Statistical significance will be set at p < 0.05.

The baseline sociodemographic and work-related characteristics of participants will be summarised using descriptive statistics. Continuous variables will be summarised according to their distribution. Normally distributed data will be presented as means with standard deviations. For variables that are not normally distributed, results will be reported as medians with interquartile ranges. Categorical variables will be described using frequencies and percentages. Baseline characteristics will be summarised separately for the intervention and comparison groups. Between-group comparability will be evaluated descriptively using absolute standardised differences rather than null-hypothesis significance tests. Characteristics showing potentially meaningful imbalance, together with prespecified potential confounders, will be considered in adjusted analyses.

GEE will be used to determine changes in outcomes across time. GEE will account for within-participant correlation arising from repeated measurements and will use all available outcome observations. Missing data and the assumptions underlying their handling are described below.Separate models will be fitted for each outcome variable, including psychological wellbeing domains, burnout, job satisfaction, absenteeism, and presenteeism.

Each model will include study group, time point, and a group by time interaction term to estimate differential changes between the intervention and comparison factories over time. Prespecified demographic or occupational covariates considered clinically or methodologically relevant will be included in adjusted models. An exchangeable working correlation structure will be specified to account for within participant correlations across repeated measurements. Link functions and distributions will be selected according to the nature of each outcome, and robust standard errors will be applied.

The GEE models will account for correlation arising from repeated observations within participants. However, because the study includes only one intervention factory and one comparison factory, reliable adjustment for factory-level clustering or estimation of cluster-robust standard errors at the factory level is not feasible. Furthermore, intervention status is completely aligned with factory. Therefore, the group-by-time interaction will be interpreted as the differential change observed between the two factories rather than as an intervention effect that is fully separable from factory-level influences.

The amount and pattern of missing data will be described for each outcome and assessment timepoint. The characteristics of participants with complete and incomplete follow-up data will be examined descriptively to identify variables potentially associated with missingness. The primary GEE analyses will use all available repeated observations. Where missing outcome or covariate data are substantial, multiple imputation using chained equations will be conducted as a sensitivity analysis under a missing-at-random assumption. The imputation model will include study group, assessment timepoint, baseline outcome values, available follow-up outcomes and variables associated with missingness. Estimates from the available-case, complete-case and imputed analyses will be compared to assess the robustness of the findings.

A per-protocol sensitivity analysis will also be conducted using a prespecified adherence criterion. Adequate adherence will be defined as attendance at the initial WMHP session and at least five of the six scheduled reinforcement sessions. This analysis will include intervention participants who received the minimum required programme exposure and comparison participants who were not exposed to any structured study-specific mental health intervention. The findings will be considered supportive and will not replace the primary intention-to-treat analysis.

### Data management and confidentiality

All data collected will be handled according to institutional data protection requirements and ethical standards. Questionnaire data will be collected using secure electronic data collection platforms. Hardcopy questionnaires will be used when there are operational constraints. The completed forms will be securely transferred into electronic databases by authorised research personnel and subsequently stored in locked cabinets. Each participant will be assigned a unique identifier to maintain confidentiality. Identifiable information will be kept separately from the research data and will not be used during data analysis.

Clinic utilisation data, MC records, and sick leave information will be obtained from panel clinic administrative records and linked to study participants using anonymised identifiers only.

All data will be retained in accordance with institutional and regulatory requirements and securely destroyed after the required retention period. Hard copy data will be kept in locked cabinets within restricted access offices. While electronic data will be securely stored on password protected computers and institutional servers accessible only to the research team.

De-identified participant-level data, the data dictionary and relevant statistical code underlying the study findings will be made available following publication, subject to participant consent, ethical requirements and applicable institutional data-governance arrangements. Where public deposition is not permitted, access to appropriately de-identified data may be considered through the relevant institutional data-access process.

### Dissemination plan

The findings of this study will be disseminated through submission to peer reviewed international journals as well as presentations at national and international conferences, including both oral and poster formats. Summary findings will be shared with participating organisations, policymakers, and relevant stakeholders. This will facilitate the decision making on WMHP adoption and expansion within manufacturing sector. Aggregated and anonymised findings may also be used to support the development of policy briefs that aimed at promoting evidence informed workplace mental health interventions.

### Ethical approval and informed consent

This study has obtained ethical approval from the University of Malaya Research Ethics Committee (UMREC) [UM.TNC2/UMREC_3983] on 19 May 2025. The Declaration of Helsinki, Malaysian Good Clinical Practice guidelines, and institutional governance standards will be adhered to in all research procedures that involve human participants.

Prior to recruitment, permission to conduct the study will be obtained from the management of each participating food manufacturing factory. Once approval is obtained, workers who meet the eligibility criteria will be given briefing sessions. During the sessions, workers will be explained on the research objectives, procedures, potential risks, expected benefits, confidentiality safeguards, and voluntary participation of the study.

Written informed consent will be obtained from the participants before any data collection. Consent forms will be in both Bahasa Melayu and English. Participation will be completely voluntary. Participants will also be informed that they may withdraw from the study at any point of time without penalty or consequences.

Participant who displays signs of severe psychological distress, including responses identified during screening, appropriate referrals will be made. Participants will be directed to available mental health services such as nearby primary care clinics or the factory’s panel clinic to ensure that adequate support is provided.

Any important protocol modifications will be submitted to the University of Malaya Research Ethics Committee for approval and updated in the trial registry where applicable. Relevant amendments will also be communicated to the participating organisations and study team.

## Discussion

This protocol outlines the design and implementation of a multi-component WMHP and its accompanying economic evaluation within food manufacturing settings in Malaysia. The study addresses an important evidence gap in workplace mental health research. Most existing evidence originates from studies conducted in high income countries and among selected occupational groups. Evidence from industrial and manufacturing settings in middle income countries remains limited [25,26,40].

Most workplace mental health intervention research has traditionally focused on service based or white-collar occupations. In contrast, researchers have paid far less attention to mental health interventions in industrial sectors, particularly within manufacturing environments. Emerging evidence indicates that workers in manufacturing environments often face substantial psychological strain linked to shift work, repetitive tasks, production pressure, and physically demanding working conditions [17,19,20]. These factors may place industrial workers at elevated risk of work-related mental health problems. Despite this, manufacturing workers remain underrepresented in intervention studies. As a result, the evidence base for workplace mental health interventions in industrial settings remains limited. By studying workers in food manufacturing factories, this research attempts to address an important gap in the literature and broaden workplace mental health evidence beyond traditional office based occupations.

Existing evidence has shown that poor mental health among workers can affect individuals, organisations and the broader economy. It is often linked to reduced productivity, higher levels of absenteeism and presenteeism, and greater usage of healthcare services [55]. Many observational studies across different occupational groups have shown that workers exposed to psychosocial stressors often experience poorer mental health. Common stressors include organisational barriers, excessive job demands, low job control, and insufficient support from supervisors. Exposure to these stressors has been linked to increased symptoms of depression, anxiety and work-related psychological distress, including burnout [9,56]. These findings highlight the need for preventive workplace interventions in strengthening workers’ coping capacity and also addressing work-related stressors.

The WMHP is developed to address key psychosocial factors affecting workers’ mental health through strategies that support stress regulation, psychological coping, and the management of work-related demands. The program integrated several approaches, namely psychoeducation, mindfulness practice, progressive muscle relaxation, and breathing exercises. Evidence demonstrates that multiple components program tends to produce better improvements in psychological outcomes than single strategy interventions [22,23]. Outcome measures will be collected at several time points before and after the intervention. This repeated assessment allows changes in psychological well-being and work-related outcomes to be observed over time. Such evaluation methods are commonly used in workplace mental health intervention research [57,58].

Beyond effectiveness, the inclusion of a CBA responds to increasing calls for economic evidence to inform employer and policy decision-making [25,59]. Existing economic evidence consistently indicates that the majority of costs associated with workplace mental health problems arise from indirect productivity losses, particularly presenteeism, rather than from direct healthcare expenditure [25,26,41,55]. Most economic evaluations have been carried out in high income countries and mainly within service sector workplaces. This limits how well the findings apply to industrial settings in middle income countries. In this study, objective information such as clinic utilisation and MC records will be analysed together with self-reported productivity outcomes. This approach aims to generate economic evidence that is relevant to employers and based on routine administrative data.

This study protocol has several strengths. The quasi-experimental design reflects the practical realities of workplace settings, where random allocation of participants is often difficult to implement [31]. The inclusion of repeated outcome measurements allows changes in psychological and work-related outcomes to be tracked over time. Such approach is consistent with longitudinal methods commonly used in workplace mental health studies [34]. The study also considers both effectiveness and economic outcomes. Evaluating these components within the same framework allows a more complete assessment of the program.

Nevertheless, several limitations should be considered. The study includes only one intervention factory and one comparison factory, resulting in complete alignment between intervention status and factory. Consequently, observed differences may partly reflect factory-level characteristics, including management practices, workforce composition, shift organisation, production demands and other contextual factors, rather than the WMHP alone. The small number of factories also prevents reliable estimation of factory-level variance or cluster-adjusted inference. Findings will therefore be interpreted as differential changes between the two participating factories and generalised cautiously. The relatively short follow-up period may also be insufficient to capture longer-term intervention effects.

Although the use of separate factories is expected to minimise direct cross-arm contamination, informal exposure of comparison participants to similar mental health information through supervisors, coworkers or other non-study sources cannot be completely excluded.

The economic evaluation is also limited by the inclusion of only one factory in each study arm. Differences in production cycles, seasonal demand, workforce composition, management practices, staffing arrangements and wider economic conditions may influence absenteeism, presenteeism and healthcare utilisation independently of the WMHP. Although the primary analysis will use incremental changes relative to the comparison factory and sensitivity analyses will apply conservative benefit-attribution assumptions, these approaches cannot fully separate programme-related benefits from factory-level influences. The resulting cost–benefit estimates will therefore be interpreted as context-specific economic findings rather than definitive causal returns attributable solely to the WMHP.

This study is expected to contribute to the field of workplace mental health in both practical and methodological ways. It assesses the economic value and effectiveness of a structured mental health program in an industrial setting. By doing so, the study aims to generate evidence that reflects real workplace conditions. The findings may offer valuable insights to policymakers, occupational health practitioners, and employers regarding the feasibility, impact, and potential ROI of workplace mental health interventions in manufacturing environments.

## Conclusion

This protocol outlines a structured framework for evaluating the WMHP implemented in food manufacturing factories in Malaysia. The study integrates both effectiveness assessment and CBA to examine the program’s overall impact. By focusing on an industrial sector that remains under-represented in current research, the study seeks to generate evidence that is relevant for policy and workplace practice. The findings are expected to support informed decision making on the adoption and long term sustainability of workplace mental health initiatives. They may also help strengthen the evidence base for integrating mental health promotion within occupational safety and health frameworks.

## Supporting information

S1 Checklist SPIRIT 2025 checklist_revised. (DOCX)

S1 File Protocol. (PDF)
