# Supplementary material for: Evaluating the effectiveness and cost–benefit of a worksite mental health program among manufacturing workers: A study protocol for a quasi-experimental study
Source: PLoS One. 2026 Oct 12;21(10):e0360481. doi: 10.1371/journal.pone.0360481 (PMC13657276; doi:10.1371/journal.pone.0360481)
Supplement: Supplementary file 1 [file pone.0360481.s002.pdf]

FORM 1: APPLICATION FORM

PART A: Brief Details of Project

1. Research Title : A COST-BENEFIT ANALYSIS ELEMENT STUDY IN DETERMINING THE EFFECTIVENESS OF A WORKSITE MENTAL HEALTH PROGRAM IN A FOOD MANUFACTURING INDUSTRY

2. Project Start : October 2024

3. Principal Investigator/Supervisor

   Name :Bala Murali A/L Sundram
   Title : Dr
   Position : Medical Lecturer
   Telephone : +60 176301906
   Email : bala.murali@um.edu.my
   Department :Department of Social and Preventive Medicine
   Academy/Faculty/Institute/Centre: Faculty of Medicine

4. Co-Investigator

   Name : Victor Hoe Chee Wei Abdullah
   Title : Dr
   Position :Lecturer
   Telephone : +60 163719146
   Email : victor@ummc.edu.my
   Department :Department of Social and Preventive Medicine
   Academy/Faculty/Institute/Centre: Faculty of Medicine

5. Co-Investigator

   Name :Lim Yin Cheng
   Title : Dr
   Position :Lecturer
   Telephone : +60 179727939
   Email : limyc@ummc.edu.my
   Department :Department of Social and Preventive Medicine
   Academy/Faculty/Institute/Centre : Faculty of Medicine

6. Co-Investigator

Name :Rama Krishna A/L Supramaniam
Title : Dr
Position :Lecturer
Telephone : +60 173625817
Email : rama.krishna@ummc.edu.my
Department :Department of Social and Preventive Medicine
Academy/Faculty/Institute/Centre : Faculty of Medicine

7. Co-Investigator (external)

Name : Amira bt Azzeri
Title : Dr
Position :Medical Officer
Telephone : +60 132088543
Email : amirah.azzeri@usim.edu.my
Department :Department of Primary Care Sciences
Academy/Faculty/Institute/Centre: Faculty of Medicine and Health Sciences, University Sains Islam Malaysia

8. Co-Investigator (external)

Name : Siti Nurani bt Hj Hassan
Title : Puan
Position : Technical expert (Consultation Research and Development (CRD) Department, National Institute of Occupational Safety and Health Malaysia (NIOSH)
Telephone : +60 192360910
Email : siti.nurani@niosh.com.my
Department :Research and Development Department, NIOSH Malaysia
Academy/Faculty/Institute/Centre: NIOSH Malaysia

9. Co-Investigator (external)

Name : Nadia bt Mohamad
Title : Dr
Position :Medical Officer
Telephone : +60 163719146
Email : nadia@moh.gov.my
Department :Occupational Health Unit, IMR
Academy/Faculty/Institute/Centre: IMR, MOH Malaysia

10. Co-Investigator (external)

Name : Kee Chee Cheong
Title : Mr
Position :Senior Research Officer
Telephone : +60 195761866

Email : kee@moh.gov.my
Department :Sector for Biostatistics and Repository, NIH
Academy/Faculty/Institute/Centre: National Institutes of Health, MOH

11. Co-Investigator (external)
Name : Raheel Nazakat
Title : Mr
Position :Senior Research Officer
Telephone : 03 3362 7610
Email : raheel@moh.gov.my
Department :Sector for Biostatistics and Repository, NIH
Academy/Faculty/Institute/Centre: National Institutes of Health, MOH

12. Co-Investigator (external)
Name : Suriya Kumareswaran
Title : Dr
Position :Senior Medical Officer of Public Health and OHD
Telephone : 010 269 7048
Email : suriya_kumareswaran@hotmail.com
Department :JKN Johor, MOH
Academy/Faculty/Institute/Centre: MOH

1. Student Principal Investigator (PI) only
Name : Yim Wei Sen
Title : Dr
Position :Medical officer
Telephone : + 60 167950804
Email : vsenyim88@gmail.com
Department :University of Malaya
Academy/Faculty/Institute/Centre : Faculty of Medicine
Degree/Programme :Doctor of Public Health (DrPH)

PART B: Data Collection

4. New data to be collected from human participant. Please tick any that apply.

☐	Focus group
☒	Experimental procedures/treatment/intervention
☐	Internet survey
☒	Observation
☐	Personal interviews
☐	Telephone survey
☒	Action research
☒	Questionnaire
☐	Others (please state):Click here to enter text.

5. Existing records with personal data.

☐	Yes
☒	No

6. Brief description of study.
   i. Background of study (less than 300words).

Occupational stress, anxiety, and burnout are prevalent issues in various industries, including the Fast- Moving Consumer Goods (FMCG) food manufacturing sector. Research indicates that these challenges have detrimental effects on employee well-being and organizational performance. Studies have shown that occupational stress and anxiety negatively impacts psychological well-being, while burnout leads to decreased job performance. Additionally, burnout can result in physical and mental health issues, such as depression and suicide. Workplace mental health programs (WMHP) have been developed to mitigate the health impacts of stress, anxiety and burnout, showing positive results in enhancing employee well-being. Workplace-related interventions regarding mindfulness, stress management, workload and time management targeting subjective perceptions of stress have been reported in the literature to have positive effects. Organizing these interventions during working hours could reduce the conflict between work and family, care work and leisure time. In general, stress management interventions can have long-term effects on anxiety and reactivity to social conflicts. It is recommended that employee engagement be included in the intervention development and implementation process. A systematic review found that person interventions for burnout tended to have short-term effects, whereas the combination of person- and organization-directed interventions achieved longer lasting effects. In Malaysia, studies on the cost-benefit analysis (CBA) of mental health prevention programs in workplaces are beginning to highlight the importance and impact of such initiatives. These studies examine the effectiveness of employee assistance programs, acceptance of mental

health off days, workplace health promotion programs, and strategies for preventing mental disorders while promoting mental health. The insights derived from these studies underscore the positive outcomes of implementing mental health programs, including improved employee well-being, reduced medical cost claims, and enhanced work engagement.

ii) Rationale of study/problem statement

The modern workplace, particularly within the Fast-Moving Consumer Goods (FMCG) food industry, presents a complex array of stressors and challenges contributing to occupational stress, anxiety and burnout. Occupational stress, anxiety and burnout within the (FMCG) food manufacturing industry have emerged as critical challenges, with significant implications for employee well-being and organizational performance. Common causes of stress in the workplace which include low pay; promotion delays; threats of redundancy; job insecurity; changes in working hours; feelings of lack of control; lack of job satisfaction; office politics; employee lack of control; career development and achievement factors; conflict at work; heavy work load; new management techniques; long hours of work; work shifts; sensory factors such as noise; sexual harassment; ageism; meeting deadlines; burn out; poor social skills; bullying at work; autocratic leadership; and concept change at work.

Mental health challenges present significant quality-of-life and financial burdens for employees and employers. Somewhere between 30% and 50% of adults, experience mental illness over their lifetimes, resulting in more than $200 billion annually in health care utilization and lost work productivity. Mental health concerns remain among the top contributors of disability worldwide. Within the workplace, acute and chronic mental health conditions result in more days of work lost and a higher likelihood of working while impaired as well as higher employee turnover. Providing appropriate mental health care to those in the workforce could help to alleviate these challenges and reduce suffering.

Cost-benefit analysis (CBA) study of a worksite mental health intervention program in the Malaysian fast-moving consumer good (FMCG) food industry would be valuable based on the available research. Implementing a WMHP program incorporated in the Employee Assistance Programs (EAP), have shown positive impacts on employee well-being These interventions have been linked to reduced sickness absenteeism, enhanced work engagement, and improved mental health among employees. In Malaysia, the cost of absenteeism and presenteeism equated to 4.5% of the GDP in 2015. This situation may provide the economic incentive to invest in human capital through workplace wellness programs to achieve a positive return of investment via increased productivity, retention of experienced employees and reduced hiring costs.

Additionally, a proposed Workplace Mental Health Program (WMHP) with stages of intervention focusing on mental health assessments, tips, and support committees has demonstrated positive outcomes on emotional well-being within organizations. Conducting a cost-benefit analysis of such programs could provide insights into the financial implications and benefits of investing in mental health interventions in the Malaysian manufacturing industry. Implementing such programs can lead to improvements in job satisfaction, performance, and outcomes, ultimately benefiting both employees and

organizations. While the initial costs of mental health interventions may be a concern, the long-term benefits in terms of improved mental health, reduced burnout, and enhanced productivity can outweigh the expenses, making it a cost-effective investment for companies in the food manufacturing industry

Objective(s) of study:

The objectives of the study are as follows:

1. To conduct a Cost Benefit Analysis (CBA) of a Workplace Mental Health Program (WMHP)
   - Establish a comprehensive list of costs and benefits associated with the WMHP program, ensuring all relevant factors are considered for a holistic analysis.
   - To determine the anticipated benefits of the program on employee well-being and organizational health.
   - Quantitatively assess the financial implications of these benefits in relation to the program's costs.
2. Determine the effectiveness of the WMHP program among the workers in the FMCG industry in Malaysia.

Study participants (new data to be collected from human participants).

i. Study sample. Please specify.

The study population will be workers involved in the production line of a FMCG food manufacturing company located in Negeri Sembilan, Malaysia. This study will include all participants in the factory, Malaysian citizens, who worked in the factory for at least one year and completed the informed consent.

The exclusion criteria will be individuals diagnosed with a psychiatric disorder and currently monitored by a psychiatrist, who have chronic diseases with major complications such as heart diseases and chronic kidney diseases, a known case of cancer, participated in the stress management program within the last 1 year and currently on medications for any psychiatric illnesses.

The sample size was estimated using an unpaired t-test via PS Software Version 3.1.2 with a 95% confidence interval, $\alpha = 0.05$. The total sample size for this study was 88 (n = 44/group). By factoring in a 10% attrition rate during the study follow up, the minimal sample size will be 97, which will be rounded up to 100(n=50/group).

ii) How will participants be recruited? Please specify.

A parallel, quasi-experimental study design will be conducted using a 1:1 allocation ratio. Participating food manufacturing factories will be identified based on willingness to participate and compiled into a sampling frame. Two factories will be selected using simple random sampling. Within each selected factory, eligible workers will be identified according to predefined inclusion and exclusion criteria. Participants will be selected using simple random sampling from the list of eligible workers provided by the human resource department, using computer-generated random numbers.

iii) Who will perform the data collection? The student PI and Co-I will perform the data collection while conducting the WMHP at the factory at Baseline, after 1 month and at 3rd Month.

Workplace Mental Health Program (WMHP) Components:

1. **StressManagement Workshops:** Conducted at Baseline, after 1 month and at 3rd Month by providing employees with knowledge and tools to manage stress, anxiety, burnout levels effectively, including time management, conflict resolution, and relaxation techniques.

- **Deep Breathing/Coherent Breathing Sessions:** Conducted at Baseline, after 1 month and at 3rd Month led by trained facilitators in the study team, focusing on teaching and practicing coherent breathing techniques to reduce stress, anxiety, burnout levels.
- **Progressive Muscle Relaxation (PMR) Sessions:** Conducted at Baseline, after 1 month and at 3rd Month led by trained facilitators in the study team, focusing on teaching and practicing PMR to reduce stress, anxiety, burnout levels.

2. **Mindfulness Training and Resilience Building activities:** Sessions incorporated into the program to enhance awareness and present-moment focus, reducing reduce stress, anxiety, burnout levels and improving emotional regulation.

3. **Questionnaires** to assess the Stress, Anxiety and Burnout levels at pre- and post-program, in the FMCG industry and determine the effectiveness of the program.

4. **Digital Resources:** Access to online materials and videos for daily stress management practice, supporting ongoing employee engagement with the program outside of structured sessions.

The PI will lead the study and collaborate with factory management to gain access to eligible workers. Information sessions will be conducted in each factory to explain the study's purpose, procedures, and benefits to potential participants.

Written informed consent from workers who agree to participate will be obtained. The consent form will detail the study objectives, data collection procedures, potential risks, and the confidentiality of responses.

The student PI will visit the factories to administer digital questionnaires during work breaks or after shifts. This ensures that participants complete the questionnaires in a controlled environment, minimizing distractions. Data will be collected at 3 time points of the WMHP, i.e., during Baseline, after 1 month and at 3rd month, by using the questionnaires after each sessions as well.

The PI and student PI will assist in the delivery of the intervention program consisting of Deep Breathing exercise, Progressive muscle relaxation techniques and Mindfulness Training and Resilience Building activities as part of the WMHP.

iii) Participant inclusion criteria

1. Full-time manufacturing factory workers at the factory in Negeri Sembilan
2. Aged between 18 and 60 years.
3. Malaysian citizens.
4. Employed for at least one year in the current factory.
5. Able to understand and respond to questionnaires in Bahasa Melayu or English.

iv) Participant exclusion criteria.

1. Individuals diagnosed with a psychiatric disorder and currently monitored by a psychiatrist, who have chronic diseases with major complications such as heart diseases and chronic kidney diseases, a known case of cancer, and currently on medications for any psychiatric illnesses.
2. Pregnant workers, as pregnancy can independently affect stress, anxiety and burnout.
3. Exclude part-time or temporary workers to maintain consistency in employment status.

v) Are the participants given any form of payment/incentive to participate?

Participants will receive an honorarium/gift as an incentive for completing the study. The honorarium will be stress balls. This incentive is provided to encourage participation and ensure completion of all required study components.

PART C: Risk and Benefits

11. Possible benefits to participants

• **Improved Mental Health and Well-being**: Participants may experience a reduction in stress, anxiety, and burnout levels due to the various mental health strategies employed in the program, such as deep breathing exercises, progressive muscle relaxation (PMR), mindfulness training, and resilience-building activities. These interventions are designed to enhance overall mental health and well-being, contributing to better emotional regulation and a more positive mental state for the FMCG industry workers.
• **Enhanced Awareness and Self-Management**: Through participation in the program, individuals will gain greater awareness of the effects of workplace stress and the importance of mental health. This knowledge empowers participants to make informed decisions about

their mental health and adopt self-management strategies that can help mitigate stress and anxiety in their daily lives.

- **Access to Support and Resources**: The program provides access to digital resources and ongoing support materials, allowing participants to continue practicing stress management techniques outside of structured sessions. This continuous access can help reinforce learning and encourage the sustained application of healthy coping strategies.
- **Increased Work Engagement and Productivity**: By addressing mental health issues, participants may see improvements in their work engagement and productivity. Reduced levels of stress and burnout can lead to increased motivation, concentration, and job satisfaction, positively impacting their performance at work.
- **Contribution to Research and Policy Development**: By participating in the program, individuals contribute to important research aimed at improving workplace mental health policies and support systems. The findings from this study could lead to better workplace environments for employees, benefiting not only the participants but also future workers in similar industries.

The **potential benefits for the company** in taking part in the Workplace Mental Health Program (WMHP) for their workers include:

- **Reduced Absenteeism and Presenteeism**: By implementing the WMHP, companies can reduce absenteeism due to stress-related illnesses and presenteeism, where employees are physically present but not fully productive due to mental health issues. This reduction in absenteeism and presenteeism can lead to significant cost savings and improve overall workforce productivity.
- **Enhanced Employee Productivity and Performance**: A focus on mental health through structured programs can lead to increased employee motivation, concentration, and engagement. As employees experience improved mental well-being, their ability to perform efficiently and effectively at work increases, enhancing overall productivity and the quality of work output
- **Improved Employee Retention and Talent Attraction**: Companies that invest in the mental well-being of their employees are more likely to retain their workforce and attract new talent. A positive workplace environment that prioritizes employee health can lead to lower turnover rates and help the company attract skilled workers who value a supportive and healthy work culture.
- **Positive Return on Investment (ROI)**: The CBA analysis helps quantify the financial benefits of the WMHP relative to its costs. A positive ROI indicates that the benefits, such as increased productivity, reduced absenteeism, and lower healthcare

costs, outweigh the program's expenses, making it a cost-effective investment for the company.

- **Compliance with Occupational Health and Safety Standards**: Participating in a WMHP aligns with best practices in occupational health and safety, helping companies comply with regulations and avoid potential legal liabilities associated with workplace mental health issues as well as for future policy directions.

12. Risk of harm (new data to be collected from human participants).

RISK	YES	NO
Will the study involve intervention, such as action research/treatment of any type? If YES, please give details:<br>The student PI and Co-I will perform the data collection while conducting the WMHP at the factory at Baseline, after 1 month and at 3rd Month. This will include administering questionnaires, training sessions on Deep Breathing exercise, Progressive muscle relaxation techniques and Mindfulness Training and Resilience Building activities as part of the WMHP; and also ensuring adherence to ethical guidelines.<br>**Workplace Mental Health Program (WMHP) Components:**<br>**StressManagement Training Workshop:** Conducted at Baseline, after 1 month and at 3rd Month by providing employees with knowledge and tools to manage stress, anxiety, burnout levels effectively, including time management, conflict resolution, and relaxation techniques.<br>• **Deep Breathing/Coherent Breathing Sessions:** Conducted at Baseline, after 1 month and at 3rd Month led by trained facilitators in the study team, focusing on teaching and practicing coherent breathing techniques to reduce stress, anxiety, burnout levels.<br>• **Progressive Muscle Relaxation (PMR) Sessions:** Conducted at Baseline, after 1 month and at 3rd Month led by trained facilitators in the study team, focusing on teaching and practicing PMR to reduce stress, anxiety, burnout levels.<br>• **Mindfulness Training and Resilience Building activities:** Sessions incorporated into the WMHP program to enhance awareness and present-moment focus, reducing reduce stress, anxiety, emotional burnout levels and improving regulation.	☒	☐

**Questionnaires** to assess the Stress, Anxiety and Burnout levels at pre- and post- program, in the FMCG industry and determine the effectiveness of the program.<br>• DASS-21 questionnaires to assess depression, anxiety and stress,<br>• Job Satisfaction Questionnaire,<br>• World Health Organization-Health and Work Performance Questionnaire (WHO-HPQ) questionnaire<br>• Oldenburg Burnout Inventory (OLBI) questionnaire<br>**Digital Resources:** Access to online materials and videos for daily stress management practice, supporting ongoing employee engagement with the program outside of structured sessions.		
Is it possible that the duration of the procedures will cause minimal stress, in particular, for children, given their age and capacity?	☐	☒
Is it possible that the study will involve greater than minimal privacy risks, which could induce stress to research participants, such as political behaviour, illegal and sexual conduct, drug or alcohol use?	☐	☒
Will the study cause psychological stress/pain/discomfort?<br>If YES, please state the precautions taken to minimize such stress/pain/discomfort/risk:	☐	☒
Are any of these participants from a minority/culturally identifiable/disadvantaged group? (e.g. *Orang Asli*)<br>Please specify: Click here to enter text.	☐	☒

i. If any of the responses above is yes, describe potential risk/conflict of interest of the study and provide a plan to mitigate the risk/conflict of interest.

To ensure the well-being of participants, the study will implement the following precautions:

Participants will receive detailed guidance and training on performing deep breathing exercise, progressive muscle relaxation techniques and mindfulness training and resilience building methods.

The research team will regularly check in with participants and provide a contact point for any concerns or discomfort.

Participants will be informed that participation is voluntary, with the option to withdraw at any time without penalty.

The study will be conducted with ethical approval, ensuring participant well-being is prioritized.

PART D: Privacy and Confidentiality

13. Describe how you will preserveparticipant's confidentiality as you collect and analyse the data and when you report theresult

Each participant will be assigned a unique identifier code, which will be used in place of their personal information on all data collection forms and during data analysis. This ensures that personal details are not directly linked to the data. All electronic data, including questionnaire responses, will be stored in secure, password-protected databases accessible only to authorized research team members.

Access to participant data will be limited to the PI and essential research team members. All team members will be trained on confidentiality protocols. Data will be de-identified before analysis, meaning that individual identities will not be known to analysts. When reporting results, data will be presented in aggregate form without any identifying information. No personal identifiers will be used in any publications or presentations resulting from the study.

If data needs to be shared with collaborators or for secondary analysis, it will be provided in an anonymized form, ensuring participants' identities remain protected. After the study is completed, data will be securely stored and then either archived securely or destroyed.

These measures ensure that participants' confidentiality is strictly maintained throughout the study process, from data collection to reporting.

14.Data record.

i. Describe briefly how the research data will be recorded, for example, audiotape, videotape or written notes. Responses from digital questionnaires will be recorded directly into a secure electronic database. A **google form** will be used to disseminate the questionnaires. Paper questionnaires, if used, will be entered into the database and then securely stored.

No Audiovisual Recording: The study will not involve the use of audiotapes or videotapes. Data collection will be limited to written and electronic formats to ensure privacy and confidentiality.

These methods ensure accurate and secure recording of research data while maintaining participant confidentiality.

Observations: The participants to be observed are full-time manufacturing factory workers in a Fast-
Moving Consumer Goods (FMCG) food manufacturing company located in Negeri Sembilan, Malaysia. These participants will include Malaysian citizens aged between 18 and 60 years, employed in the factory for at least one year and meeting the inclusion criteria. The **outcomes measured** by participating in the **Worksite Mental Health Program**
(WMHP) and the questionnaires in the study include both **psychological and physiological outcomes** that are critical for evaluating the program's effectiveness. Based on the study design, the following outcomes are being measured.

1. Psychological Outcomes:

- **Stress Levels:**
  - Measured using the **DASS-21 (Depression, Anxiety, and Stress Scale)**, which assesses participants' stress levels before and after the intervention.
- **Anxiety Levels:**
  - Anxietyisalso measured using the **DASS-21** questionnaire.
- **Burnout Levels:**
  - Measuredusing the **Oldenburg Burnout Inventory (OLBI)**, which assesses both emotional exhaustion and disengagement, core aspects ofburnout.
- **Job Satisfaction:**

- Assessed using the **Job Satisfaction Questionnaire**, which gauges participants' satisfaction with theirwork environment and duties before and after the intervention.

- **Work Productivity and Impairment:**
  - Measured through the **World Health Organization-Health and Work Performance Questionnaire (WHO-HPQ)** questionnaire, which looks at absenteeism, presenteeism, and overall work impairment related to health issues.

2. Program Effectiveness:

- **Cost-Benefit Analysis (CBA) Outcomes:**
  - This study also aims to conduct a **Cost-Benefit Analysis (CBA)** to measure the financial benefits of the program, such as reduced absenteeism, presenteeism, and improved work engagement, compared to the cost of implementing the mental health program.

A written informed consent will be obtained from all participants before the study begins. The consent form will clearly explain the study's objectives, procedures, and the non- invasive nature of the observation. It will also assure participants that their personal data will be kept confidential.

The observation during the program will be strictly non-invasive, meaning that participants will not be subjected to any intrusive measures that may alter their normal behaviour or cause discomfort. The goal is to observe their natural engagement in the WMHP activities without introducing any external factors that might influence their responses.

There will be no physical contact or invasive monitoring devices applied to participants. There will be no interference with the participants' normal activities outside the program sessions, and no additional stress or discomfort will be imposed.

The observation will focus on how participants engage with the mental health activities, particularly:

- Their **attendance and participation** in stress management workshops and mindfulness exercises.
- Their **level of involvement** in activities such as deep breathing, PMR, and resilience-building exercises.
- Any **observable changes in demeanour**, such as increased relaxation or attentiveness during session

The program has been designed to be a **supportive and beneficial** experience for participants, with the aim of reducing stress, anxiety, and burnout. The observation process aligns with this goal by being unobtrusive and designed to minimize any additional pressure on participants. Participants will be reminded that their participation is voluntary, and they have the right to withdraw from the observation or the study at any point without penalty.

Observationswillbe recorded and computed into an excel sheet in a password protected personal laptop-questionnaires (google form).Allobservations measurements will be anonymized to protect participants'identities. The investigators will not collect names or other identifying informationduringthe observation sessions, and all data will be stored securely and confidentially.Noaudio-visual recordings will be made to protect participants' privacy. All data will beanonymized, and no identifiable information, such as names or photographs, will be linked tothedata. The research team will strictly adhere to ethical guidelines, ensuring that no harm,stigma, or psychological discomfort arises from the observation process.

ii. Describe what you will do with the recorded data once it has been analysed.
All extracted data will be entered and saved on a password-protected laptop. Participants’ names will be de-identified and linked with unique identification numbers. No identifiers will be used on participant data sheets. All data will be kept confidential and not disclosed to any third party. Upon completion of study, any hardcopy data will be stored in a locked office of the principal investigator and maintained for a minimum of seven years after the completion of the study. The hardcopy data will be destroyed after that period of storage.

iii. Specify who apart from yourself will have access to the research data.
All investigators may have access to the research data.

iv. Details who will own the data and the results of your research.

The data and the results are owned by the investigators.

PARTE: Conflict of Interest

15.Do any of the researchers have any potential conflicts of interest?

The investigators declare they have no conflict of interest.

PART F: Attachments

Please tick the boxes- which of the following documents are enclosed.

☒	Questionnaire/ interview script
☒	Participant Information Sheet
☒	Informed Consent Form
☐	Others : Please state :______________________________

FORM 2: PARTICIPANT INFORMATION SHEET

Research title:

A COST-BENEFIT ANALYSIS ELEMENT STUDY IN DETERMINING THE EFFECTIVENESS OF A WORKSITE MENTAL HEALTH PROGRAM IN A FOOD MANUFACTURING INDUSTRY

Researchers:

No	Name of Personnel (inclusive of owner & principal)	Position & Responsibility	Contact No
1.	Dr Bala Murali Sundram	Senior Medical Lecturer (PI)	bala.murali@um.edu.my 017 6301906
2.	Dr Lim Yin Cheng	Senior Medical Lecturer	limyc@ummc.edu.my
3.	Dr Victor Hoe Chee Wei Abdullah	Professor	victor@ummc.edu.my
4.	Dr Rama Krishna Supramaniam	Senior Medical Lecturer	rama.krishna@ummc.edu.my
5.	Dr. Amirah binti Azzeri	Senior Lecturer Community Health Unit, Department of Primary Care	amirah.azzeri@usim.edu.my
6.	Dr Nadia Mohamad	Senior Medical Officer, Occupational Health Unit, NIH, MOH	nadia@moh.gov.my
7.	En Raheel Nazakat	Senior Researcher, NIH, MOH	raheel@moh.gov.my
8.	Mr Kee Chee Cheong	Senior Researcher and Biostatistician, NIH, MOH	kee@moh.gov.my
9.	Dr. Suriya Kumareswaran	Senior Medical Officer	suriya_kumareswaran@hotmail.com
10.	Pn Siti Nurani bt Hj Hassan	NIOSH Technical Expert (Mental Health and Wellness Trainer), Consultation Research and Development, NIOSH	siti.nurani@niosh.com.my

11.	Dr Yim Wei Sen	Doctorate student	vsenyim88@gmail.com

Introduction:

The research is being conducted by a team led by Dr. Bala Murali Sundram, a Senior Medical Lecturer and Occupational and Public Health Medicine Specialist at the University of Malaya. The research team includes experienced professionals in public health and occupational safety who are working together to evaluate the effectiveness of a workplace mental health program in the food manufacturing industry. Their role is to design and implement the study, analyze the data collected, and assess the cost-benefit of the mental health program to improve employee well-being and productivity. The findings from this research will help develop better mental health support systems in the workplace and inform policy decisions aimed at improving working conditions.

Purpose:

The purpose of this study is to conduct a cost-benefit analysis (CBA) of the WMHP program, a worksite mental health intervention. We aim to determine the effectiveness of this program in reducing sickness absenteeism, improving work engagement levels, and decreasing medical cost claims among workers in the food manufacturing industry.

Study Procedure:

Participants will be involved in various activities, including completing surveys and participating in mental health intervention sessions. These sessions will include deep breathing exercises, progressive muscle relaxation (PMR), mindfulness training, stress management workshops, and resilience-building activities. Data will be collected on sickness absenteeism, medical cost claims, and work engagement levels through surveys and records provided by the Human Resource's department. The study will last for approximately 12 months. Participants will be required to complete baseline assessments, engage in the intervention program, and participate in follow-up assessments after three months of completing the intervention.

Participation in the Study:

You have been identified as a potential participant because you work in the food manufacturing industry, and your input is valuable in understanding the effects of the mental health program. Participation is entirely voluntary, and you are free to withdraw from the study at any time without any impact on your employment or any other adverse consequence.

Benefit to participants:

- By participating in this study, you will gain access to a comprehensive mental health program designed to help manage stress, anxiety, and burnout. You will receive feedback on your mental health and well-being, which may help you better understand and manage your health. Additionally, you will contribute to important research aimed at improving workplace conditions and mental health support systems. Participants will receive an honorarium- stress ball as an incentive for completing the study. This incentive is provided to encourage participation and ensure completion of all required study components.

Risk to participants:

- While there are no significant risks associated with participating in this study, some activities may cause slight discomfort due to the discussion of personal stress, anxiety and burnout levels. If at any point you feel distressed or require assistance, support services will be available, and we will provide you with information on how to access these services. Their concerns will be discussed thoroughly and suggested for appropriate referral pathway to the in-house clinic of the factory or the nearest healthcare centre should escalation of care be required.

Confidentiality:

- All information collected during this study will remain confidential. Data will be stored securely and will only be accessible to the research team. Your data will be anonymized, and no personal identifiers will be used in any reports or publications resulting from this study. Audio or video recordings, if made, will be used solely for research purposes and will be destroyed after the study is completed.

Complaints

Should the participants have any concerns or questions about the research project, which they do not wish to discuss with the researchers listed in the document, then they may contact:

Reviewing REC	University of Malaya Research Ethics Committee (UMREC)
Telephone	03-79677022 (ext: 2369)
Email	umrec@um.edu.my
Mailing address	Pusat Perkhidmatan Penyelidikan (PPP)Level 2, Kompleks Pengurusan Penyelidikan dan Inovasi (KPPI)University of Malaya 50603, Kuala Lumpur, Malaysia

By clicking “I agree” below you are indicating that you are at least 18 years old, have read and understood this consent form and agree to participate in this research study.

☐ Agree

☐ Disagree

FORM 3: CONSENT FORM

(ENGLISH)

Title of research:

To become a participant in the research, you or your parent/legal guardian are advised to sign this

Consent Form.

I herewith confirm that I have met the requirement of age and am capable of acting on behalf of myself /* as a parent/legal guardian as follows:

1. I understand the nature and scope of the research being undertaken.
2. All my questions relating to this research and my participation therein have been answered to my satisfaction.
3. I voluntarily agree to take part in this research, to follow the study procedures and to provide all necessary information to the investigators as requested.
4. I may at any time choose to withdraw from this research without giving reasons.
5. I have received a copy of the Subjects Information Sheet and Consent Form.
6. Except for damages resulting from negligent or malicious conduct of the researcher(s), I hereby release and discharge University of Malaya and all participating researchers from all liability associated with, arising out of, or related to my participation and agree to hold them harmless from any harm or loss that may be incurred by me due to my participation in the research.
7. I have read and understood all the terms and conditions of my participation in the research.

I have read the statements above, understand the same, and voluntarily sign this form.
Dated :_____ day _____ month ______ year

Name of participants (18 years & above): ____

IC/Passport number: ___________________________________

Signature: ___________________________ Date:

To be filled by parents/legal guardians for participants aged below 18 years

Name of parent/legal guardian: ____

IC/Passport number Parent/legal guardian: __________________________________

Signature of parent/legal guardian: _______________ Date:____________________

Ralationship of the guardian to the participant:_______________________________________

Name of researcher: ___ ____ ______ ____ __________ IC/Passport number: __________________

Signature of researcher: ________________________

SARINGAN MINDA SIHAT

Nama :

I/C No. :

Jantina :

Umur :

Bangsa :

Pekerjaan :

No. Telefon :

Tarikh :

Ceraikan keratan ini untuk disimpan oleh klien.

KEPUTUSAN SARINGAN MINDA SIHAT

Nama : Tarikh :

Jantina : Lelaki/Perempuan Umur :

Ujian	Keputusan		
	Stres	Anzieti	Kemurungan
DASS			

SARINGAN MINDA SIHAT

SOAL SELIDIK DASS

Langkah 1 : Sila baca dan jawab soal selidik DASS.

Langkah 2 : Masukkan skala markah jawapan ke dalam ruangan kosong dibahagian 2, mengikut soalan (S) bagi setiap kategori (Stres, Anzieti dan Kemurungan).

Langkah 3 : Jumlahkan skala markah bagi setiap kategori bagi mengetahui tahap status kesihatan mental anda.

Langkah 4 : Sila isikan keputusan dalam bahagian 3 dan isikan dalam keratan di muka hadapan.

BAHAGIAN 1

Sila baca setiap kenyataan di bawah dan **bulatkan** jawapan anda pada kertas jawapan berdasarkan jawapan **0, 1, 2 atau 3** bagi menggambarkan keadaan anda sepanjang minggu yang lalu. Tiada jawapan yang betul atau salah. Jangan mengambil masa yang terlalu lama untuk menjawab mana-mana kenyataan.

*Please read each statement and **circle** number **0, 1, 2 or 3** which indicates how much the statement applied to you over the past week. There are no right or wrong answers. Do not spend too much time on any statement.*

0 = **Tidak Langsung** menggambarkan keadaan saya
__Did not__ apply to me at all

1 = **Sedikit atau jarang-jarang** menggambarkan keadaan saya
*Applied to me to some **degree, or some of the time***

2 = **Banyak atau kerapkali** menggambarkan keadaan saya
*Applied to me to a **considerable degree**, or a **good part of time***

3 = **Sangat banyak atau sangat kerap** menggambarkan keadaan saya
*Applied to me **very much, or most of the time***

1.	Saya dapati diri saya sukar ditenteramkan<br>*I found it hard to wind down*	0	1	2	3
2.	Saya sedar mulut saya terasa kering<br>*I was aware of dryness of my mouth*	0	1	2	3
3.	Saya tidak dapat mengalami perasaan positif sama sekali<br>*I couldn't seem to experience any positive feeling at all*	0	1	2	3
4.	Saya mengalami kesukaran bernafas (contohnya pernafasan yang laju, tercungap-cungap walaupun tidak melakukan senaman fizikal)<br>*I experienced breathing difficulty (eg, excessively rapid breathing, breathlessness in the absence of physical exertion)*	0	1	2	3
5.	Saya sukar untuk mendapatkan semangat bagi melakukan sesuatu perkara<br>*I found it difficult to work up the initiative to do things*	0	1	2	3
6.	Saya cenderung untuk bertindak keterlaluan dalam sesuatu keadaan<br>*I tended to over-react to situations*	0	1	2	3
7.	Saya rasa menggeletar (contohnya pada tangan)<br>*I experienced trembling (eg, in the hands)*	0	1	2	3
8.	Saya rasa saya menggunakan banyak tenaga dalam keadaan cemas<br>*I felt that I was using a lot of nervous energy*	0	1	2	3
9.	Saya bimbang keadaan di mana saya mungkin menjadi panik dan melakukan perkara yang membodohkan diri sendiri<br>*I was worried about situations in which I might panic and make a fool of myself*	0	1	2	3
10.	Saya rasa saya tidak mempunyai apa-apa untuk diharapkan<br>*I felt that I had nothing to look forward to*	0	1	2	3
11.	Saya dapati diri saya semakin gelisah<br>*I found myself getting agigate*	0	1	2	3
12.	Saya rasa sukar untuk relaks<br>*I found it difficult to relax*	0	1	2	3
13.	Saya rasa sedih dan murung<br>*I felt down-hearted and blue*	0	1	2	3
14.	Saya tidak dapat menahan sabar dengan perkara yang menghalang saya meneruskan apa yang saya lakukan<br>*I was intolerant of anything that kept me from getting on with what I was doing*	0	1	2	3
15.	Saya rasa hampir-hampir menjadi panik/cemas<br>*I felt I was close to panic*	0	1	2	3
16.	Saya tidak bersemangat dengan apa jua yang saya lakukan<br>*I was unable to become enthusiastic about anything*	0	1	2	3
17.	Saya rasa tidak begitu berharga sebagai seorang individu<br>*I felt I wasn't worth much as a person*	0	1	2	3
18.	Saya rasa saya mudah tersentuh<br>*I felt that I was rather touchy*	0	1	2	3
19.	Saya sedar tindakbalas jantung saya walaupun tidak melakukan aktiviti fizikal (contohnya kadar denyutan jantung bertambah, atau denyutan jantung berkurangan)<br>*I was aware of the action of my heart in the absence of phycial exertion (eg, sense of heart rate increase, heart missing a beat*	0	1	2	3
20.	Saya berasa takut tanpa sebab yang munasabah<br>*I felt scared without any good reason*	0	1	2	3
21.	Saya rasa hidup ini tidak bermakna<br>*I felt that life was meaningless*	0	1	2	3

BAHAGIAN 2

Panduan Mengira Skor :-
Masukkan skala markah jawapan bagi soalan (S) bagi setiap kategori.

STRES								
Soalan	S1	S6	S8	S11	S12	S14	S18	Jumlah
Markah								

ANZIETI								
Soalan	S2	S4	S7	S9	S15	S19	S20	Jumlah
Markah								

KEMURUNGAN (DEPRESSION)								
Soalan	S3	S5	S10	S13	S16	S17	S21	Jumlah
Markah								

Selepas dijumlahkan, sila rujuk kepada petak skor saringan dan terjemahkan jumlah skor untuk mengetahui tahap status kesihatan mental anda.

SKOR SARINGAN			
	Kemurungan	Anzieti	Stres
Normal	0 - 5	0 - 4	0 - 7
Ringan	6 - 7	5 - 6	8 - 9
Sederhana	8 - 10	7 - 8	10 - 13
Teruk	11 - 14	9 - 10	14 - 17
Sangat Teruk	15 +	11 +	18 +

BAHAGIAN 3

Isikan keputusan **(normal, ringan, sederhana, teruk atau sangat teruk)** dalam jadual di bawah.

KEPUTUSAN UJIAN DASS

Ujian	Tahap
Stres	
Anzieti	
Kemurungan	

SKOR DASS	

JOB SATISFACTION QUESTIONNAIRE

Appendix 1

Please read each statement carefully and determine the degree to which you agree with these statements below. Kindly take your organisation into consideration as you respond to each statement. Please mark only 1 answer for each statement.

Sila baca setiap penyataan di bawah dengan teliti dan tentukan tahap sejauh mana anda bersetuju terhadap penyataan berikut. Sila pertimbangkan organisasi anda dalam menjawab setiap penyataan. Sila tanda hanya 1 jawapan bagi setiap penyataan.

A. Teamwork (TW) / *Kerja Berpasukan*

	Strongly disagree/ *Sangat tidak setuju*	Disagree/ *Tidak setuju*	Uncertain/ *Tidak pasti*	Agree/*Setuju*	Strongly agree/ *Sangat setuju*
1. My co-workers are committed to doing quality work. *Rakan sekerja saya komited untuk melakukan kerja yang berkualiti.*	1	2	3	4	5
2. It is easy to get along with my colleagues. *Adalah sangat mudah untuk bergaul dengan rakan sekerja saya.*	1	2	3	4	5
3. I feel part of a team in working towards shared goals. *Saya merasakan saya berada di dalam satu pasukan dalam mencapai matlamat kerja yang sama.*	1	2	3	4	5
4. I experience a spirit of cooperation in this organisation. *Saya dapat merasakan semangat bekerjasama di dalam organisasi ini.*	1	2	3	4	5
5. I receive assistance from co-workers when necessary. *Saya mendapat bantuan dari rakan sekerja bila diperlukan.*	1	2	3	4	5

B. Leadership (LD) / *Kepimpinan*

	Strongly disagree/ *Sangat tidak setuju*	Disagree/ *Tidak setuju*	Uncertain/ *Tidak pasti*	Agree/*Setuju*	Strongly agree/ *Sangat setuju*
1. My supervisor/senior manager visibly demonstrates a commitment to quality. *Penyelia saya menunjukkan komitmen dalam menghasilkan kerja yang berkualiti.*	1	2	3	4	5
2. It is clear to me what my supervisor expects of me regarding my job performance. *Penyelia saya menerangkan dengan jelas mengenai harapan beliau berkenaan prestasi kerja saya.*	1	2	3	4	5
3. My supervisor is able to address my questions or concerns. *Penyelia saya dapat menangani soalan atau kebimbangan saya.*	1	2	3	4	5
4. My supervisor has strong management skills. *Penyelia saya mempunyai kemahiran pengurusan yang bagus.*	1	2	3	4	5

C. Reward and Recognition (RR) / *Ganjaran dan Pengiktirafan*

	Strongly disagree/ *Sangat tidak setuju*	Disagree/ *Tidak setuju*	Uncertain/ *Tidak pasti*	Agree/*Setuju*	Strongly agree/ *Sangat setuju*
1. My base pay is fair for my responsibilities. *Gaji pokok saya adalah berpatutan untuk pekerjaan saya.*	1	2	3	4	5
2. The annual raise is reasonable. *Kadar kenaikan gaji tahunan saya adalah munasabah.*	1	2	3	4	5
3. I am satisfied with the retirement plan. *Saya berpuas hati dengan pelan persaraan saya.*	1	2	3	4	5
4. The process used to determine promotions/annual raises is just and fair. *Proses kenaikan gaji tahunan dan kenaikan pangkat adalah adil dan saksama.*	1	2	3	4	5
5. I received the right amount of recognition or praise for work that is well done. *Saya menerima penghargaan yang sewajarnya untuk pencapaian cemerlang saya.*	1	2	3	4	5

D. Empowerment and Participation (EP) / *Penguasaan dan Penyertaan dalam Organisasi*

	Strongly disagree/ *Sangat tidak setuju*	Disagree/ *Tidak setuju*	Uncertain/ *Tidak pasti*	Agree/*Setuju*	Strongly agree/ *Sangat setuju*
1. I understand the vision of my organisation. *Saya memahami visi organisasi saya.*	1	2	3	4	5
2. The mission and purpose of my organisation make me feel that my job is important. *Misi dan matlamat organisasi membuatkan saya merasakan pekerjaan saya sangat penting.*	1	2	3	4	5
3. I feel I've contributed to the organisation's plan and mission. *Saya merasakan saya menyumbang kepada rancangan dan misi organisasi.*	1	2	3	4	5
4. My job makes good use of my skills and abilities. *Kerja saya memanfaatkan kemahiran dan kebolehan saya.*	1	2	3	4	5
5. I am satisfied with my involvement in decisions that affect my work. *Saya berpuas hati dengan penglibatan saya dalam membuat keputusan di dalam pekerjaan saya.*	1	2	3	4	5

E. Training and Individual Development (TD) / *Latihan dan Perkembangan Individu*

	Strongly disagree/ *Sangat tidak setuju*	Disagree/ *Tidak setuju*	Uncertain/ *Tidak pasti*	Agree/ *Setuju*	Strongly agree/ *Sangat setuju*
1. My initial training provided by the hospital was sufficient. *Latihan/kursus awal yang diberikan oleh pihak hospital kepada saya adalah mencukupi.*	1	2	3	4	5
2. As much ongoing training I need is provided by the Hospital. *Pihak hospital menyediakan latihan/kursus berterusan yang diperlukan oleh saya.*	1	2	3	4	5
3. Training offered by the organisation helps me to be effective and efficient in my job. *Latihan/kursus yang ditawarkan membantu saya menjadi lebih cemerlang dan cekap dalam kerja.*	1	2	3	4	5
4. The organisation encourages continued education and professional growth. *Organisasi ini menggalakkan pembelajaran berterusan dan perkembangan dalam kerjaya.*	1	2	3	4	5
5. I have opportunities at work to learn and grow. *Saya diberi peluang di tempat kerja untuk belajar dan mengembangkan kerjaya saya.*	1	2	3	4	5

F. Working Hours (WH) / *Masa Bekerja*

	Strongly disagree/ *Sangat tidak setuju*	Disagree/ *Tidak setuju*	Uncertain/ *Tidak pasti*	Agree/ *Setuju*	Strongly agree/ *Sangat setuju*
1. I am satisfied with my total working hours. *Saya berpuas hati dengan jumlah waktu bekerja saya.*	1	2	3	4	5
2. I am given the flexibility in scheduling my working hours. *Saya diberikan kelonggaran dalam mengatur waktu bekerja saya.*	1	2	3	4	5
3. I have the flexibility to manage my work and nonwork interests e.g. caring responsibilities, study, sports interests, etc. *Saya diberi kelonggaran dalam menguruskan perkara yang berkaitan ataupun tidak berkaitan dengan pekerjaan saya seperti sukan, pembelajaran dan tanggungjawab lain.*	1	2	3	4	5

G. Communication (C) / *Komunikasi*

	Strongly disagree/ *Sangat tidak setuju*	Disagree/ *Tidak setuju*	Uncertain/ *Tidak pasti*	Agree/*Setuju*	Strongly agree/ *Sangat setuju*
1. This organisation does an excellent job of keeping employees informed about matters affecting us. *Organisasi ini telah berjaya menyampaikan informasi yang tepat berkenaan sesuatu perkara.*	1	2	3	4	5
2. Management clearly explains the reasons behind decisions on key issues. *Pihak pengurusan menyatakan sebab yang jelas di sebalik setiap keputusan berkaitan isu-isu utama.*	1	2	3	4	5
3. I know where to go within the organisation to obtain information that I need. *Saya mengetahui tempat rujukan di dalam organisasi saya untuk mendapatkan maklumat yang saya perlukan.*	1	2	3	4	5
4. My supervisor clearly communicates his/her expectations of my job performance. *Penyelia saya menyampaikan harapannya tentang prestasi kerja saya dengan jelas.*	1	2	3	4	5

H. Working Condition (WC) / *Persekitaran Kerja*

	**Strongly disagree/ *Sangat tidak setuju***	**Disagree/ *Tidak setuju***	**Uncertain/ *Tidak pasti***	**Agree/ *Setuju***	**Strongly agree/ *Sangat setuju***
1. My physical working conditions (lighting, ventilation, ergonomic, etc) are good. *Persekitaran kerja saya (pencahayaan, pengudaraan, ergonomik, dan lain-lain) sangat bagus.*	1	2	3	4	5
2. I have the tools and resources that I need in order to do my work right. *Saya mempunyai sumber dan alatan yang mencukupi untuk melakukan pekerjaan saya.*	1	2	3	4	5
3. The amount of work expected of me is reasonable. *Jumlah beban kerja saya adalah berpatutan.*	1	2	3	4	5

Supplement I: The Full Oldenburg Burnout Inventory - Malay Version (OLBI-M)

Malay (translated version)	English (original version)
1. Saya sentiasa mencari aspek yang baru dan menarik dalam pekerjaan saya.<br>1) Sangat setuju<br>2) Setuju<br>3) Tidak setuju<br>4) Sangat tidak setuju	1. I always find new and interesting aspects in my work.<br>1) Strongly agree<br>2) Agree<br>3) Disagree<br>4) Strongly disagree
2. Ada hari-hari tertentu saya berasa letih sebelum sampai di tempat kerja (seperti pejabat, kelas dan sebagainya).<br>1) Sangat setuju<br>2) Setuju<br>3) Tidak setuju<br>4) Sangat tidak setuju	2. There are days when I feel tired before I arrive at work.<br>1) Strongly agree<br>2) Agree<br>3) Disagree<br>4) Strongly disagree
3. Saya seringkali bercakap tentang kerja saya secara negatif.<br>1) Sangat setuju<br>2) Setuju<br>3) Tidak setuju<br>4) Sangat tidak setuju	3. It happens more and more often that I talk about my work in a negative way.<br>1) Strongly agree<br>2) Agree<br>3) Disagree<br>4) Strongly disagree
4. Selepas kerja saya memerlukan lebih banyak masa berbanding dahulu untuk berasa tenang dan lebih baik.<br>1) Sangat setuju<br>2) Setuju<br>3) Tidak setuju<br>4) Sangat tidak setuju	4. After work, I tend to need more time than in the past in order to relax and feel better.<br>1) Strongly agree<br>2) Agree<br>3) Disagree<br>4) Strongly disagree
5. Saya boleh menangani tekanan kerja dengan sangat baik.<br>1) Sangat setuju<br>2) Setuju<br>3) Tidak setuju<br>4) Sangat tidak setuju	5. I can tolerate the pressure of my work very well.<br>1) Strongly agree<br>2) Agree<br>3) Disagree<br>4) Strongly disagree
6. Akhir-akhir ini, saya kurang berfikir sewaktu kerja dan hampir melakukan kerja secara automatik.<br>1) Sangat setuju<br>2) Setuju<br>3) Tidak setuju<br>4) Sangat tidak setuju	6. Lately, I tend to think less at work and do my job almost mechanically.<br>1) Strongly agree<br>2) Agree<br>3) Disagree<br>4) Strongly disagree
7. Saya mendapati pekerjaan saya merupakan suatu cabaran yang positif.<br>1) Sangat setuju<br>2) Setuju<br>3) Tidak setuju<br>4) Sangat tidak setuju	7. I find my work to be a positive challenge.<br>1) Strongly agree<br>2) Agree<br>3) Disagree<br>4) Strongly disagree
8. Semasa kerja, saya kerap kali berasa kekosongan dari segi emosi.<br>1) Sangat setuju<br>2) Setuju<br>3) Tidak setuju<br>4) Sangat tidak setuju	8. During my work, I often feel emotionally drained.<br>1) Strongly agree<br>2) Agree<br>3) Disagree<br>4) Strongly disagree

(*continued on next page*)

Supplement I: (*Continued*)

Malay (translated version)	English (original version)
9. Dengan masa berlalu, saya menjadi terasing daripada pekerjaan ini.<br>1) Sangat setuju<br>2) Setuju<br>3) Tidak setuju<br>4) Sangat tidak setuju	9. Over time, one can become disconnected from this type of work.<br>1) Strongly agree<br>2) Agree<br>3) Disagree<br>4) Strongly disagree
10. Selepas bekerja, saya mempunyai tenaga yang mencukupi untuk aktiviti masa lapang.<br>1) Sangat setuju<br>2) Setuju<br>3) Tidak setuju<br>4) Sangat tidak setuju	10. After working, I have enough energy for my leisure activities.<br>1) Strongly agree<br>2) Agree<br>3) Disagree<br>4) Strongly disagree
11. Kadang-kadang saya berasa menyampah dengan kerja saya.<br>1) Sangat setuju<br>2) Setuju<br>3) Tidak setuju<br>4) Sangat tidak setuju	11. Sometimes I feel sickened by my work tasks.<br>1) Strongly agree<br>2) Agree<br>3) Disagree<br>4) Strongly disagree
12. Selepas kerja, saya kebiasaannya berasa lesu dan penat.<br>1) Sangat setuju<br>2) Setuju<br>3) Tidak setuju<br>4) Sangat tidak setuju	12. After my work, I usually feel worn out and weary.<br>1) Strongly agree<br>2) Agree<br>3) Disagree<br>4) Strongly disagree
13. Saya hanya boleh bayangkan jenis kerja ini sahaja yang boleh saya buat.<br>1) Sangat setuju<br>2) Setuju<br>3) Tidak setuju<br>4) Sangat tidak setuju	13. This is the only type of work that I can imagine myself doing.<br>1) Strongly agree<br>2) Agree<br>3) Disagree<br>4) Strongly disagree
14. Kebiasaannya, saya boleh mengurus jumlah kerja saya dengan baik.<br>1) Sangat setuju<br>2) Setuju<br>3) Tidak setuju<br>4) Sangat tidak setuju	14. Usually, I can manage the amount of my work well.<br>1) Strongly agree<br>2) Agree<br>3) Disagree<br>4) Strongly disagree
15. Saya berasa semakin sibuk dengan pekerjaan saya.<br>1) Sangat setuju<br>2) Setuju<br>3) Tidak setuju<br>4) Sangat tidak setuju	15. I feel more and more engaged in my work.<br>1) Strongly agree<br>2) Agree<br>3) Disagree<br>4) Strongly disagree
16. Apabila bekerja, saya selalu rasa bertenaga.<br>1) Sangat setuju<br>2) Setuju<br>3) Tidak setuju<br>4) Sangat tidak setuju	16. When I work, I usually feel energised.<br>1) Strongly agree<br>2) Agree<br>3) Disagree<br>4) Strongly disagree

Appendix – Eight questions from the locally validated, short Malay version of World Health Organization-Health and Work Performance Questionnaire

1. What is your current monthly salary (inclusive of allowances)?

   Berapakah jumlah pendapatan bulanan anda (termasuk elaun) sekarang?

2. Has a doctor ever informed you that you had a new chronic disease for the past eight years? If yes, choose whichever is applicable (Bronchial asthma/ chronic obstructive pulmonary disease, diabetes mellitus, chronic kidney disease, migraine, others – to specify)

   Pernahkah doktor beritahu anda bahawa anda menghidapi penyakit kronik baru sepanjang jangka masa lapan tahun yang lepas? Jika ya, sila pilih yang berkaitan (Asma/ penyakit paru-paru obstruktif kronik, kencing manis, penyakit buah pinggang, migrain, lain-lain – sila nyatakan)

3. Has a doctor ever informed you that you had a new acute disease for the past one month? If yes, choose whichever is applicable [Upper respiratory tract infection, acute gastroenteritis, motor vehicle accident, coronavirus disease (COVID-19), pregnancy, others – to specify]

   Pernahkah doktor beritahu anda bahawa anda menghidapi penyakit akut baru sepanjang jangka masa satu bulan yang lepas? Jika ya, sila pilih yang berkaitan [Jangkitan saluran pernafasan atas, selesema perut,, kemalangan jalan raya,, penyakit koronavirus 2019 (COVID-19), kehamilan, lain-lain – sila nyatakan]

4. How many hours does your employer expect you to work in a typical 7-day week?

Berapakah jumlah jam yang diminta oleh majikan anda untuk bekerja dalam tempoh 7 hari seminggu yang biasa?

5. In the past 4 weeks (28 days), how many days did you

   Sepanjang 4 minggu (28 hari) yang lalu, berapa harikah anda habiskan dengan

   a. Miss an entire work day because of problems with cardiovascular disease (CVD)?

      Tidak masuk bekerja sepanjang hari kerana masalah berkaitan dengan penyakit kardiovaskular?

   b. Miss an entire work day for any other reason (including vacation)?

      Tidak masuk bekerja sepanjang hari kerana sebab lain (termasuk bercuti)?

   c. Miss part of a work day because of problems with CVD?

      Tidak masuk bekerja sebahagian hari kerana masalah berkaitan dengan penyakit kardiovaskular?

   d. Miss part of a work day for any other reason (including vacation)?

      Tidak masuk bekerja sebahagian hari kerana sebab lain (termasuk bercuti)?

6. About how many hours altogether did you work in the past 4 weeks (28 days)?

   Berapakah jumlah jam anda bekerja sepanjang 4 minggu yang lalu (28 hari)?

7. On a scale from 0 to 10 where 0 is the worst job performance anyone could have at your job and 10 is the performance of a top worker, how would you rate the usual performance of most workers in a job similar to yours?

Dengan menggunakan skala 0 hingga 10 di mana 0 adalah prestasi kerja terburuk dan 10 adalah prestasi kerja terbaik, bagaimanakah anda menilai prestasi kerja kebanyakan pekerja lain yang melakukan pekerjaan yang sama atau hampir sama dengan anda?

8. Using the same 0 to 10 scale, how would you rate your overall job performance on the days you worked during the past 4 weeks (28 days)?

   Dengan menggunakan skala 0 hingga 10 yang sama, bagaimanakah anda menilai prestasi kerja anda sendiri pada hari anda bekerja sepanjang 4 minggu yang lalu (28 hari)?
